# A chemigenetic indicator based on a synthetic chelator and a green fluorescent protein for imaging of intracellular sodium ions†

**DOI:** 10.1039/d4cb00256c

**Published:** 2024-12-03

**Authors:** Shiori Takeuchi, Shosei Imai, Takuya Terai, Robert E. Campbell

**Affiliations:** a Department of Chemistry, Graduate School of Science, The University of Tokyo 7-3-1 Hongo Bunkyo-ku Tokyo 113-0033 Japan terai@chem.s.u-tokyo.ac.jp campbell@chem.s.u-tokyo.ac.jp; b CERVO Brain Research Center and Department of Biochemistry, Microbiology, and Bioinformatics, Université Laval, Québec Québec G1V 0A6 Canada

## Abstract

A chemigenetic indicator with an affinity suitable for imaging of intracellular sodium ions (Na^+^) in mammalian cells was developed. The indicator, based on a chimera of green fluorescent protein (GFP) and HaloTag labeled with a synthetic crown ether chelator, was produced by a combination of rational design and directed evolution. In mammalian cells the indicator exhibited an approximately 100% increase in excitation ratio when the cells were treated with 20 mM Na^+^ and an ionophore.

## Abstract 2

We report the development of a chemigenetic fluorescent sodium ion indicator with an affinity optimized for imaging physiologically relevant sodium ion concentrations in mammalian cells.

## Graphical abstract

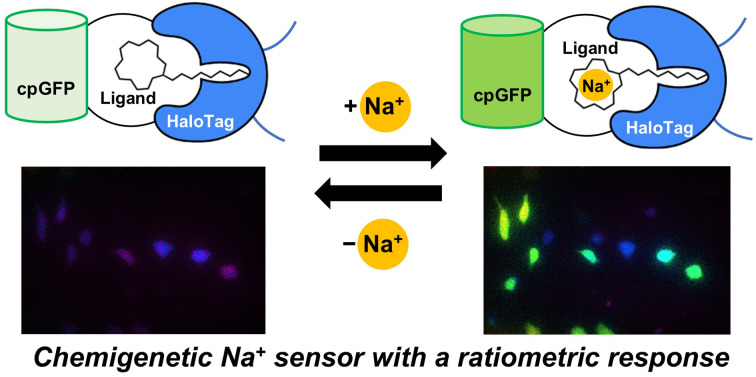

## Introduction

Na^+^ has many important biological roles. In most tissues, it is the most abundant extracellular metal ion with a concentration of about 150 mM (whereas potassium ion (K^+^) is ∼4 mM). In the intracellular milieu, the Na^+^ concentration is about 15 mM (whereas K^+^ is ∼150 mM). A Na^+^ concentration of 18 mM was measured for the cytosol of HEK293T cells, rising to 25 mM during metabolic inhibition.^1^ Resting Na^+^ concentrations of 18 mM and 9 mM were estimated for cockroach salivary duct cells and acinar peripheral cells, respectively, rising to 34 mM and 21 mM, respectively, upon stimulation with dopamine.^2^ The concentration gradient across the cellular membrane is maintained by Na^+^/K^+^-ATPases^3^ and enables various processes including kidney function and propagation of neuronal action potentials.^4^ Pathological imbalances in the Na^+^ and K^+^ concentration gradient are associated with hypertension and a variety of other disorders.^5^

To obtain insight into the spatiotemporal dynamics of Na^+^ in tissues, researchers can use Na^+^-responsive fluorescent dye-based indicators.^6^ An early example of such an indicator is sodium-binding benzofuran isophthalate (SBFI) which employs a 15-crown-5-ether as a chelator for Na^+^.^7^ While widely used, a drawback of SBFI is possible photocytotoxicity arising from its excitation wavelengths in the ultraviolet (UV) region. Later examples of Na^+^ indicators that can be excited in the visible region include Sodium Green,^8^ CoroNa Green,^9^ CoroNa Red,^10^ and ANG-2,^2^ all featuring 15-crown-5-ether derivatives. However, these later examples all exhibit intensiometric fluorescent responses which are less well-suited to quantitative imaging. SBFI exhibits an excitation ratiometric response that facilitates quantitative imaging due to being insensitive to differences in indicator concentration, such as may occur when there are changes in cell morphology due to changes in osmotic pressure.

Genetically encoded protein-based biosensors are another important class of tools for biological fluorescence imaging.^11^ Typically they are a genetic chimera of a fluorescent protein (FP) and a target-binding protein that undergoes a substantial conformational change. Compared with synthetic indicators, their advantages include near-ideal biocompatibility, targeted localization in cells (or organelles) of interest, and the ability to be iteratively optimized by directed protein evolution.^12,13^ The archetypical example is the GCaMP series of calcium ion (Ca^2+^) biosensors which have arguably replaced synthetic dye-based Ca^2+^ indicators as the tool of choice for most biological imaging applications.^14^ Beyond Ca^2+^, the specificity scope of genetically-encoded biosensors has been expanded to K^+^ and Zn^2+^,^15,16^ anions,^17,18^ metabolites,^19^ membrane voltage,^20^ enzyme activities,^21^ and other targets of interest.^22^ However, there have been no reports of a fully protein-based Na^+^ biosensor because there is no known Na^+^-binding protein that undergoes a suitably large conformational change. Generally, the specificity scope of fully genetically-encoded biosensors is strictly limited by the availability of appropriate target-binding proteins.

**Fig. 1:**
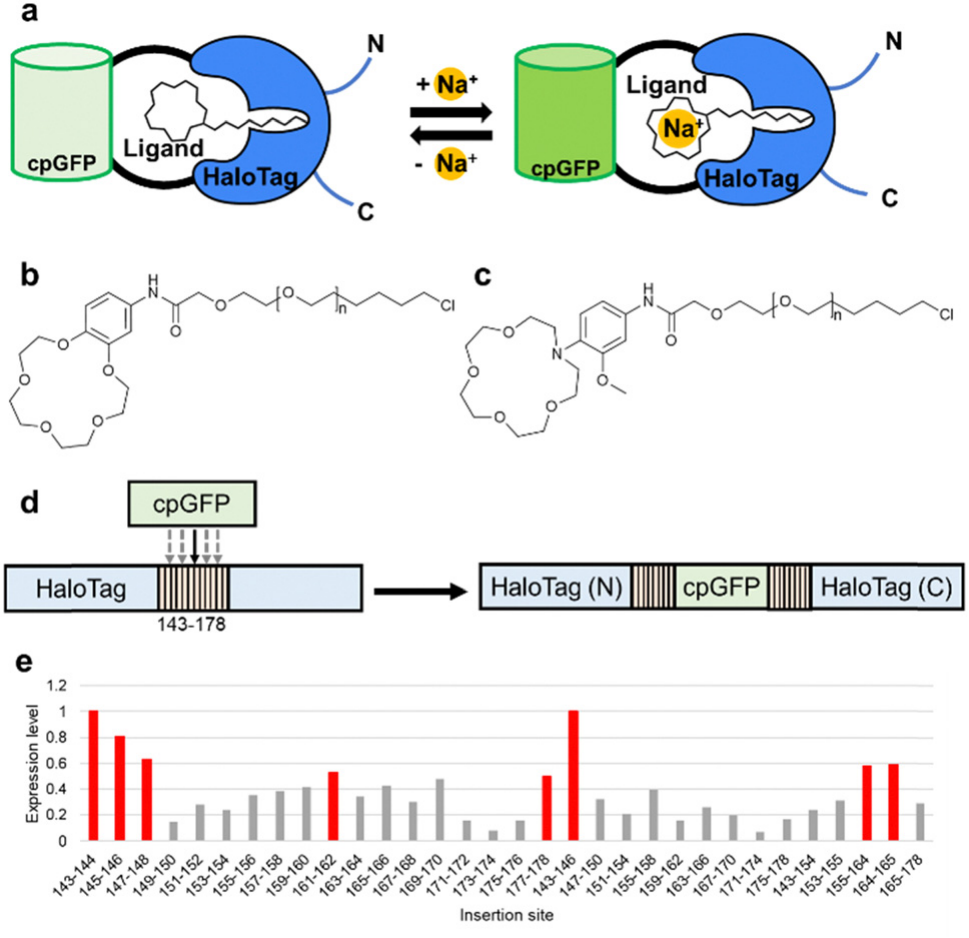
(a) Schematic diagram of the HaloGFP-Na indicator. (b) The structure of the BCE-HTL__*n*__ synthetic ligand previously used with HaloGFP-Na0.5 (*n* = 2). (c) The structure of the ACE-HTL_*n*_ synthetic ligand used in this work. (d) Schematic representation of HaloGFP, which was created from HaloTag and cpGFP. GFP was inserted at 32 different sites in the range of residue numbers 143–178 of HaloTag. (e) Expression levels of 32 HaloGFPs normalized by the expression level of HaloGFP with cpGFP inserted at site 143–144. Variants that had more than half of the expression of HaloGFP with cpGFP inserted at site 143–144 are labelled in red.

In an effort to broaden the scope of genetically-encoded biosensors to include specificities for which no appropriate target-binding protein exists, we recently introduced an indicator design in which a protein-based chimera is augmented with a synthetic ion chelator (Fig. 1a).^23^ In this chemigenetic strategy, we engineered genetic chimeras of a green FP (GFP) and the self-labeling HaloTag protein (designated as HaloGFPs).^24^ By labeling of a HaloGFP chimera with synthetic HaloTag ligands (HTL; a chloroalkane) linked to appropriate chelating motifs for Ca^2+^ (*i.e.*, BAPTA) and Na^+^ (*i.e.*, benzo-15-crown-5-ether (BCE-HTL_*n*_, where *n* = 0–4; Fig. 1b)), we were able to create ion-specific indicators. Relative to the alternative approach of directly conjugating a synthetic Na^+^ indicator to HaloTag,^25^ this strategy has several advantages. One advantage is the lack of background fluorescence from the non-specific staining that can arise when cells are treated with a synthetic fluorophore. A second advantage is that the HaloGFP-based indicators are amenable to directed evolution for improved properties that could in principle include brightness, affinity, and specificity.

The mechanism of these indicators likely involves a chelation-dependent conformational change in the protein that alters the GFP chromophore environment such that there is a change in fluorescence intensity. This change could be due to a change in quantum yield, extinction coefficient, or the equilibrium between the neutral (dim; absorbance ∼400 nm) and anionic (bright; absorbance ∼500 nm) protonation states of the chromophore.^26^ A change between the chromophore protonation states can result in a change in the excitation ratio. The Ca^2+^ indicator (HaloGFP-Ca1.0 treated with the optimal BAPTA ligand) exhibited an emission intensiometric Δ*F*/*F*_0_ of 9.2 in response to Ca^2+^, and was demonstrated to function in mammalian cell cultures, including primary neurons. The Na^+^ indicator (HaloGFP-Na0.5 protein treated with the optimal BCE-HTL_2_; Fig. 1b) exhibited an excitation ratiometric Δ*R*/*R*_0_ of 2.9 for 400 mM Na^+^ and an apparent *K*_d_ for Na^+^ of 96 mM (in the absence of K^+^). As the apparent *K*_d_ was much greater than typical intracellular Na^+^ concentrations (∼15 mM), HaloGFP-Na0.5 + BCE-HTL_2_ was not suitable for imaging of physiologically relevant intracellular Na^+^ concentrations (Fig. S1, ESI†).

In the present work we aimed to overcome the limitations of our previously reported HaloGFP-Na0.5 + BCE-HTL_2_ and create a new iteration of the chemigenetic Na^+^ indicator with an effective *K*_d_ for Na^+^ that is closer to the typical intracellular concentration.

## Results and discussion

To develop a higher affinity chemigenetic Na^+^ indicator, we took inspiration from the structures of synthetic Na^+^ indicators for intracellular imaging.^2,7,8^ Specifically, we redesigned the Na^+^-chelator to be an aza-15-crown-5-ether (ACE) with a pendent phenyl group that has a methoxy group at the ortho position and the HaloTag-reactive chloroalkane at the para position (ACE-HTL_*n*_, where *n* = 0 to 4; Fig. 1c). This type of chelator is reported to have higher affinity, possibly due to the methoxy group serving as an additional axial substituent for a Na^+^ chelated within the aza-15-crown-5-ether ring.^7,27–29^ The series of five ACE-HTL_*n*_ ligands were synthesized in a three-step procedure (Fig. S2, ESI†).

With the ACE-HTL_*n*_ ligands in hand, each was used to treat the purified protein component (HaloGFP-Na0.5) of our previously chemigenetic Na^+^ indicator.^23^ Unfortunately, none of the five HaloGFP-Na0.5 + ACE-HTL_*n*_ constructs exhibited a measurable Na^+^-dependent change in fluorescence (Fig. S3, ESI†). This negative result was not unexpected, since the HaloGFP-Na0.5 protein was the product of seven rounds of directed evolution that specifically aimed to optimize its function with BCE-HTL_2_. Apparently the structural differences between the ACE and BCE chelators was sufficient to disrupt the established response mechanism.

In an effort to develop an improved iteration of the HaloGFP-Na indicator design, we repeated many of the same steps that we had originally used to arrive at HaloGFP-Na0.5 + BCE-HTL_2_, but we used the ACE-HTL_*n*_ ligands instead of the BCE-HTL_*n*_ ligands. The first step was to identify the most promising combination of HaloGFP variant and ACE-HTL_*n*_ ligand. We had previously reported a series of 32 HaloGFP variants in which cpGFP was inserted into various locations (and with replacement of various number of residues) in solvent exposed regions (residues 143–178 of HaloTag protein) close to the ligand-attachment site (Fig. 1d).^30^ Building upon past experience, we used three criteria to identify the most promising prototype: HaloGFP protein expression level; Na^+^-dependent fluorescence change with 500 nm excitation; and *K*_d_ for Na^+^. In our previous work we had independently identified the 143–144 insertion site (that is, HaloTag with residues 143–144 replaced with cpGFP) as the most promising starting point for engineering both the Ca^2+^ and Na^+^ indicators.^23^ Notably, this is also the protein with one of the highest expression levels in *E. coli*, suggesting that higher protein stability and folding efficiency might be an important consideration. With this consideration in mind, we determined that eight out of 32 HaloGFP proteins accumulated to at least half the concentration of the 143–144 variant when expressed in *E. coli* (Fig. 1e and Table S1, ESI†).

Each of the eight highest expressing HaloGFPs were treated with each of the five ACE-HTL_*n*_ ligands (40 combinations total), and their fluorescence changes in response to 25 mM Na^+^ (at 500 nm excitation) were determined (Table S2, ESI†). In addition, each of the 40 combinations was treated with 0 mM, 25 mM, 50 mM, 100 mM, and 200 mM NaCl and an approximate apparent *K*_d_ value was calculated. By both criteria, the combination of the 164–165 insertion site (that is, HaloTag with residues 164–165 replaced with cpGFP) and the ACE-HTL_1_ ligand (that is, the ligand shown in Fig. 1c with *n* = 1) gave the best performance (Tables S2 and S3, ESI†). Specifically this combination, designated HaloGFP-Na2.0 + ACE-HTL_1_, exhibited an apparent *K*_d_ of 22 mM and a Δ*F*/*F*_0_ of 0.13 (Δ*F*/*F*_0_ = (*F* − *F*_0_)/*F*_0_ where *F* is the fluorescence intensity at a particular [Na^+^] and *F*_0_ is the fluorescence intensity at [Na^+^] = 0 mM) for 25 mM Na^+^ (Tables S2 and S3, ESI†). In response to a saturating concentration of 200 mM Na^+^ (and no K^+^), HaloGFP-Na2.0 + ACE-HTL_1_ had a Δ*F*/*F*_0_ of 0.76 and an excitation ratiometric change of 0.42 (Δ*R*/*R*_0_ = (*R* − *R*_0_)/*R*_0_, where *R* is defined as *R* = fluorescence at 555 nm with 500 nm excitation divided by fluorescence at 555 nm with 400 nm excitation) at a particular [Na^+^] and *R*_0_ is *R* at [Na^+^] = 0 mM). In the presence of 100 mM K^+^ the response to 200 mM Na^+^ decreased to Δ*F*/*F*_0_ = 0.27 and Δ*R*/*R*_0_ = 0.18 (Fig. S4, ESI†).

As we had done in our previous work,^23^ we next turned to directed protein evolution to further increase the Na^+^-dependent fluorescence response of HaloGFP-Na2.0 + ACE-HTL_1_. We performed four iterative rounds of library creation. In each round a template gene (with an N-terminal 6 × His tag) was used as the template for error prone PCR or site-saturation mutagenesis to create a library of randomly mutated variants. This library was expressed in *E. coli* and approximately 400 bright green fluorescent colonies were picked, cultured, pelleted, and lysed with B-PER (Pierce). The clarified bacterial lysate was treated with ACE-HTL_1_ and the fluorescence excitation spectra were measured ± Na^+^ (0 mM, 10 mM, 50 mM). Variants (∼10 each round) that exhibited the largest intensiometric or ratiometric changes were re-expressed in *E. coli*, Ni-NTA purified, and subjected to a more extensive set of titration-based characterization, including one with Na^+^ alone (0–150 mM), one with K^+^ alone (0–150 mM), and one with 0–200 mM Na^+^ in the presence of 100 mM K^+^. The variant that was found to have the optimal compromise of high affinity for Na^+^, high response to Na^+^, and low affinity for K^+^, was used as the template for the next round of directed evolution.

**Fig. 2:**
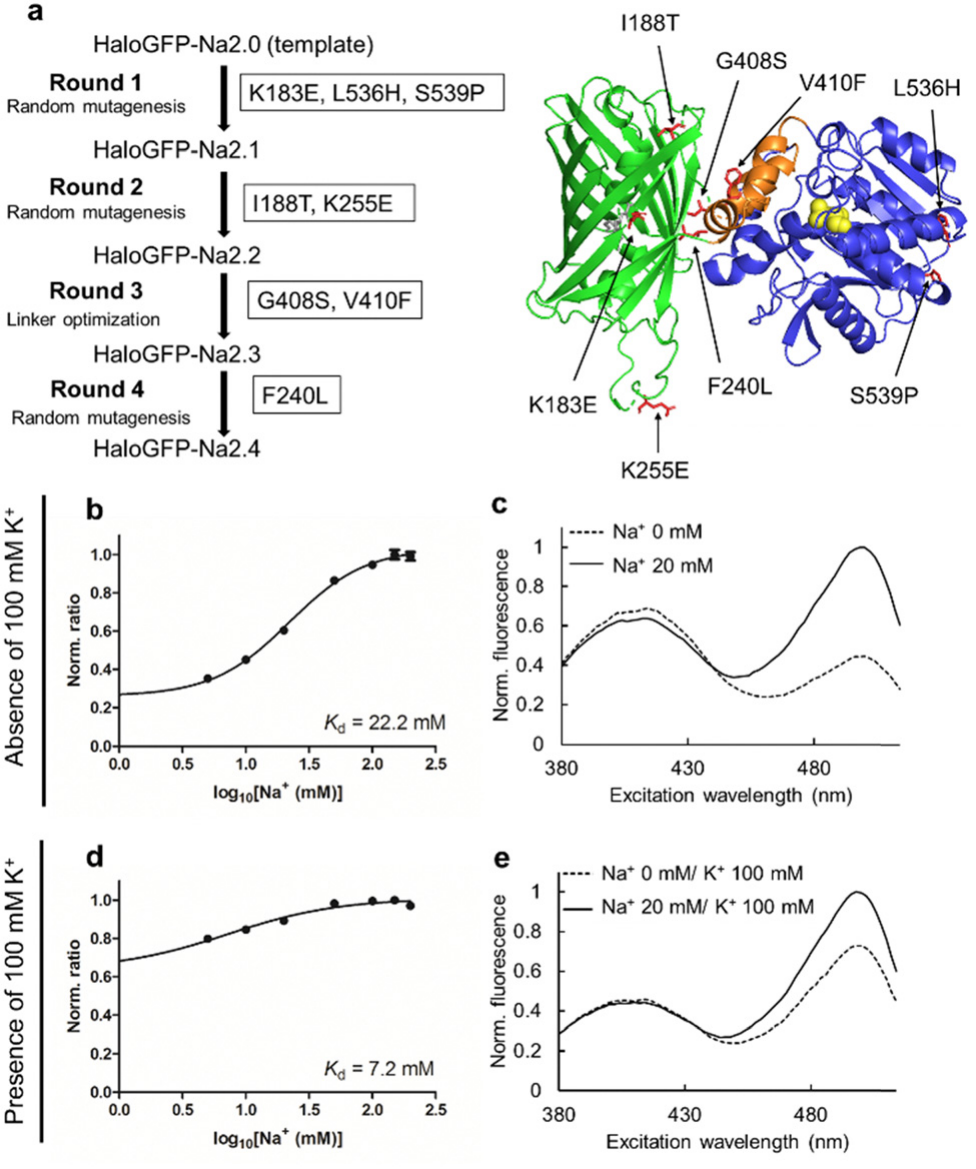
(a) Summary of mutations acquired during four rounds of directed evolution. The structure is predicted by AlphaFold2.^31^ (b) and (c) Excitation ratio as a function of Na^+^ concentration (b) and fluorescence excitation spectra (555 nm emission wavelength) of HaloGFP-Na2.4 + ACE-HTL_1_ ± 20 mM Na^+^, in the absence of 100 mM KCl (c). The spectra for (b) were measured with the ligand (*n* = 1) in the absence of 100 mM KCl in 30 mM Tris–HCl (pH 7.2) at the indicated Na^+^ concentrations at 400 nm and 500 nm excitation wavelength and 555 nm emission wavelength. The error bar shows ± S.D. (*N* = 3). (d) and (e) Excitation ratio as a function of Na^+^ concentration (d) and fluorescence excitation spectra of HaloGFP-Na2.4 + ACE-HTL_1_ ± 20 mM Na^+^, in the presence of 100 mM KCl (e). Other than the presence of KCl, all other conditions were identical to those described above. The error bar shows ± S.D. (*N* = 3).

The top variant identified in the fourth round of directed evolution (in combination with ACE-HTL_1_) was designated HaloGFP-Na2.4 + ACE-HTL_1_. DNA sequencing revealed that HaloGFP-Na2.4 had eight amino acid substitutions relative to HaloGFP-Na2.0 (Fig. 2a and Fig. S5, ESI†). HaloGFP-Na2.4 + ACE-HTL_1_ exhibited a *K*_d_ for Na^+^ of 22 mM (Fig. 2b). Spectroscopic characterization revealed a Na^+^-dependent increase in the 500 nm peak, and a small decrease in the 400 nm peak, in both the fluorescence excitation and absorbance spectra (Fig. 2c and Fig. S6, ESI†). The quantum yield of the 500 nm peak did not change in response to Na^+^. In contrast, the quantum yield of the 400 nm peak slightly increased (Table S4, ESI†). The maximum excitation ratiometric response (Δ*R*/*R*_0_) was 2.9 at 150 mM Na^+^ (Fig. 2b). The Δ*R*/*R*_0_ was 1.4 in response to 20 mM Na^+^ (Fig. 2b and c). Control experiments without the addition of ACE-HTL_1_ confirmed that the full response was ligand dependent (Fig. S7 and S8, ESI†). HaloGFP-Na2.4 + ACE-HTL_1_ exhibited an apparent p*K*_a_ of 8.8 in the Na^+^-free state, and 7.5 in the Na^+^-bound state, when excited at 500 nm (Fig. S9, ESI†). A similar shift in p*K*_a_ was observed for the HaloGFP-Na2.4 without ACE-HTL_1_ (Fig. S9, ESI†), although the fluorescence change was smaller. *In vitro* measurements of the kinetics of ligand labeling with purified protein revealed that the protein was essentially fully labeled within about 90 minutes (Fig. S10, ESI†).

These results suggest that the main response mechanism of HaloGFP-Na2.4 + ACE-HTL_1_ is a Na^+^-dependent modulation of the GFP chromophore p*K*_a_, causing the equilibrium to shift away from the dim 400 nm neutral (*i.e.*, protonated) state and towards the 500 nm bright anionic (i.e, deprotonated) state. Notably, the expected decrease at 400 nm is more evident in the absorbance spectrum (Fig. S6, ESI†) than in the excitation spectrum (Fig. 2c) due to the increased quantum yield of the 400 nm peak in the Na^+^-bound state (Table S4, ESI†).

HaloGFP-Na2.4 with ACE-HTL_1_ gave similar ratiometric responses to 20 mM Na^+^ and 150 mM K^+^, but effectively no response to biologically relevant concentrations of other cations (Fig. S11a, ESI†). Further characterization of purified HaloGFP-Na2.4 + ACE-HTL_1_ in the presence of 100 mM K^+^ revealed a *K*_d_ for Na^+^ of 7.2 mM (Fig. 2d), an excitation Δ*R*/*R*_0_ = 0.39 at 20 mM Na^+^, and a maximum Δ*R*/*R*_0_ = 0.56 at 150 mM Na^+^ (Fig. 2d and e). These diminished responses of HaloGFP-Na2.4 + ACE-HTL_1_ in the presence of 100 mM K^+^ are consistent with K^+^ competing with Na^+^ for binding to the crown ether-based chelator, as has been previously observed.^32^ No other biologically relevant cations were found to substantially compete for Na^+^ binding (Fig. S11b, ESI†). Indeed, a K^+^ titration in the absence of Na^+^ revealed a Δ*R*/*R*_0_ = 0.58 at 20 mM K^+^ and a *K*_d_ for K^+^ of 43 mM (Fig. S12, ESI†), indicating only a modest specificity for Na^+^ over K^+^. Nevertheless, the fact that we successfully achieved physiologically relevant *K*_d_ values that were substantially lower than that of HaloGFP-Na0.5 + BCE-HTL_2_, validates the design rationale of the ACE-HTL_1_ ligand.

**Fig. 3:**
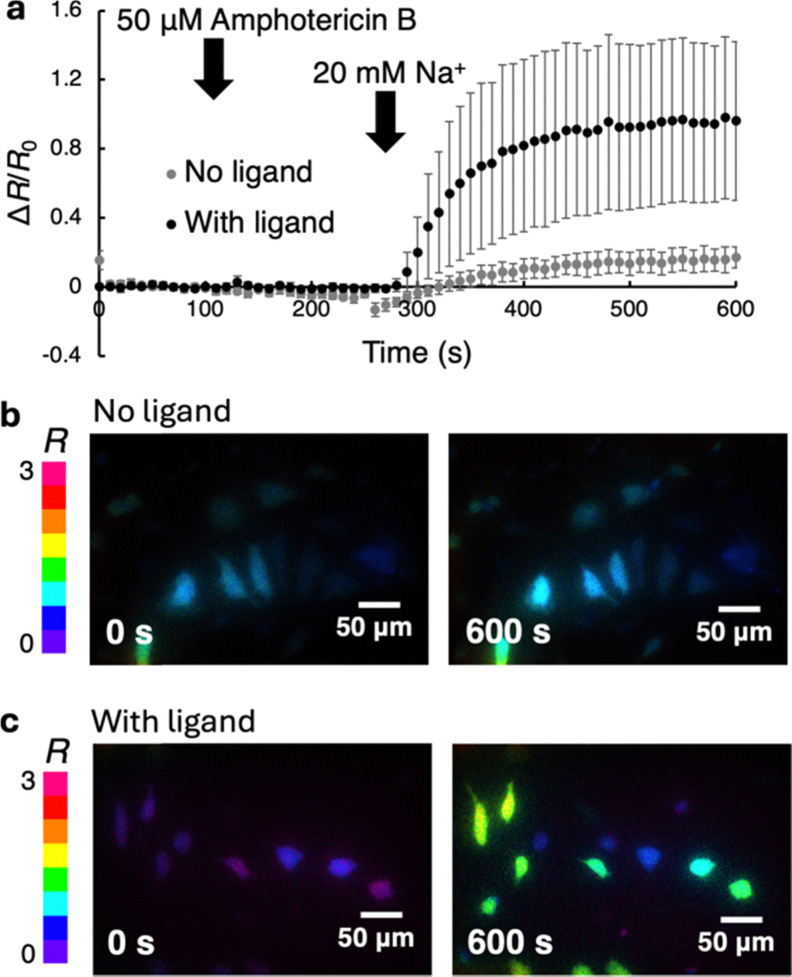
(a) Ratiometric responses to Na^+^ treatment for permeabilized HeLa cells expressing HaloGFP-Na2.4 and incubated with or without the ACE-HTL_1_ ligand. The error bar shows ± S.D. (*N* = 11 with ligand, *N* = 10 without ligand). (b) and (c) Ratiometric images of permeabilized HeLa cells used to acquire the data shown in (a). Shown in (b) are cells that are expressing HaloGFP-Na2.4, but have not been treated with ACE-HTL_1_, before (left image) and after (right image) Na^+^ addition. Shown in (c) are cells that are expressing HaloGFP-Na2.4 and have been treated with ACE-HTL_1_, and have been otherwise treated identically to the cells in (b). The color bar shows the color representations for ratios ranging from 0 to 3.

To demonstrate the applicability of HaloGFP-Na2.4 + ACE-HTL_1_ in cells, we performed mammalian cell imaging under non-physiological conditions. HeLa cells were transfected with the plasmid encoding HaloGFP-Na2.4. At 48–72 hours after transfection, cells were incubated with and without ACE-HTL_1_ for 90 minutes. Cells were washed with Na^+^-free imaging buffer containing 145 mM of *N*-methyl-d-glucamine, which is used to maintain extracellular ionic strength. GFP fluorescence was excited with two separate wavelengths, 470 nm and 405 nm, and the ratiometric change was monitored. The Na^+^ ionophore amphotericin B was added to permeabilize the cell membrane toward Na^+^, and then 20 mM Na^+^ was added. For cells containing HaloGFP-Na2.4 + ACE-HTL_1_, the excitation ratio was observed to increase by approximately 100% (Fig. 3a and c). In the absence of ACE-HTL_1_, no substantial increase in ratio was observed (Fig. 3a and b). The response was substantially larger than that obtained for HaloGFP-Na0.5 + BCE-HTL_2_ in cells that were treated identically (Fig. S13, ESI†). These results indicated that HaloGFP-Na2.4 + ACE-HTL_1_ could be used to detect at least artificially large changes in Na^+^ concentration in live cells.

## Conclusions

In summary, we have succeeded in developing a chemigenetic Na^+^ indicator (HaloGFP-Na2.4 + ACE-HTL_1_) that combines a synthetic chelator and a fluorescent protein and has sufficiently high affinity to be used for intracellular Na^+^ imaging. This indicator exhibits an excitation ratiometric change of about 40% in response to 20 mM Na^+^ even in the presence of 100 mM K^+^*in vitro*. Notably, this work also demonstrates how it is possible to optimize the performance of this HaloGFP-type design of chemigenetic indicator through the combined use of protein engineering and structural tuning of the synthetic moiety.

Despite the fact that it has a near-ideal Na^+^ affinity, HaloGFP-Na2.4 + ACE-HTL_1_ is unlikely to be broadly useful for biological Na^+^ imaging due to its limited fluorescence response. Physiologically-relevant changes in intracellular Na^+^ concentration are generally between a resting concentration of 10 to 20 mM and a stimulated maximum of 20–30 mM.^1,2^ For such a change, HaloGFP-Na2.4 + ACE-HTL_1_ would only give a fraction of the response shown in Fig. 3. However, we are optimistic that further exploration of HaloTag insertion sites, further directed evolution, and further synthetic tuning of the chelator moiety, will result in indicators with higher fluorescence response and higher Na^+^ selectivity. Yet another future direction could be to extend this design to other colors of fluorescent protein, alternative self-labeling proteins, and a broader range of ion-chelating moieties. Ultimately, this design could provide a palette of versatile indicators for multicolor and multi-analyte biological imaging.

## Data availability

The data underlying this article are available in the article and in the ESI.† Bacterial and mammalian cell expression plasmids for HaloGFP-Na2.4 are available from Addgene (#228470 and #228471, respectively).

## Conflicts of interest

There are no conflicts to declare.

## Supplementary Material

CB-OLF-D4CB00256C-s001
