# Supplementary material for: A chemigenetic indicator based on a synthetic chelator and a green fluorescent protein for imaging of intracellular sodium ions
Source: RSC Chem Biol. 2024 Dec 3;6(2):170–4. doi: 10.1039/d4cb00256c (PMC11638762; doi:10.1039/d4cb00256c)
Supplement: CB-006-D4CB00256C-s001 [file CB-006-D4CB00256C-s001.pdf]

Supplementary Information (SI) for RSC Chemical Biology.
This journal is © The Royal Society of Chemistry 2024

Electronic Supplementary Information

A chemigenetic indicator based on a synthetic chelator and a green fluorescent protein for imaging of intracellular sodium ions

Shiori Takeuchi,[a] Shosei Imai,[a] Takuya Terai,[a]* Robert E. Campbell[a,b]*

[a] Department of Chemistry, Graduate School of Science, The University of Tokyo, 7-3-1 Hongo, Bunkyo-ku, Tokyo 113-0033, Japan.

[b] CERVO Brain Research Center and Department of 863 Biochemistry, Microbiology, and Bioinformatics, Université 864 Laval, Québec, Québec G1 V 0A6, Canada.

E-mail: terai@chem.s.u-tokyo.ac.jp (T.T.); campbell@chem.s.u-tokyo.ac.jp (R.E.C.)

Supplementary Tables

Table S1. Relative expression levels of HaloGFP variants.

Insertion site	Protein concentration (µM)	Relative expression level
143-144	11.48	1
145-146	9.3	0.81
147-148	7.28	0.63
149-150	1.68	0.15
151-152	3.17	0.28
153-154	2.77	0.24
155-156	4.05	0.35
157-158	4.32	0.38
159-160	4.82	0.42
161-162	6.07	0.53
163-164	3.92	0.34
165-166	4.98	0.43
167-168	3.43	0.3
169-170	5.53	0.48
171-172	1.88	0.16
173-174	0.9	0.08
175-176	1.88	0.16
177-178	5.77	0.5
143-146	11.53	1
147-150	3.67	0.32
151-154	2.4	0.21
155-158	4.52	0.39
159-162	1.88	0.16
163-166	3	0.26
167-170	2.32	0.2
171-174	0.83	0.07
175-178	1.92	0.17
143-154	2.78	0.24
153-155	3.58	0.31
155-164	6.62	0.58
164-165	6.8	0.59
165-178	3.32	0.29

Table S2. Fluorescence changes ($\Delta F/F_0$) in response to 25 mM Na^+ for selected combinations of chloroalkanes (**ACE-HTL$_n$**) and HaloGFP proteins. Excitation wavelength is 500 nm. Combinations that showed over 10 % change are highlighted in red.

**Insertion site**	**no ligand**	**n = 0**	**n = 1**	**n = 2**	**n = 3**	**n = 4**
143-144	-0.052	-0.031	-0.018	0.031	0.031	-0.066
145-146	**0.132**	**0.111**	0.061	0.03	0.031	0.002
147-148	-0.039	-0.049	-0.027	-0.031	-0.008	-0.024
161-162	-0.036	-0.016	0	0.012	0.002	0.004
177-178	-0.023	0.029	-0.004	0.005	0.013	0.007
143-146	-0.029	0.01	-0.088	-0.137	-0.039	-0.018
155-164	0.02	0.014	0.001	0.041	0.023	0.037
164-165	-0.004	0.007	**0.128**	0.017	0.036	0.048

Table S3. K_d values for 40 combinations of chloroalkanes (**ACE-HTL$_n$**) and HaloGFP proteins. Combinations that gave values of less than 30 mM of K_d are highlighted in red.

**Insertion site**	**no ligand**	**n = 0**	**n = 1**	**n = 2**	**n = 3**	**n = 4**
143-144	over 200	over 200	over 200	157.9	35.8	174.2
145-146	over 200	47.4	48	49.5	over 200	ND
147-148	ND	ND	ND	**24.8**	ND	ND
161-162	ND	ND	ND	ND	ND	85.9
177-178	44.5	ND	49.6	32.2	ND	ND
143-146	37	80.5	185.5	62.7	142	186.3
155-164	over 200	172.9	over 200	over 200	60.2	over 200
164-165	195	ND	**22.4**	**26**	50	**23.2**

Table S4. Quantum yield of HaloGFP-Na2.4 measured at the excitation wavelengths of 400 nm and 480 nm in the absence or presence of Na^+ (0 mM NaCl or 100 mM NaCl in 30 mM Tris-HCl at pH 7.2) and with and without **ACE-HTL$_1$**. The concentration of HaloGFP-Na2.4 was 5 μM and that of **ACE-HTL$_1$** was 10 μM.

	**With ACE-HTL$_1$**		**Without ACE-HTL$_1$**	
Excitation wavelength (nm)	400 nm	480 nm	400 nm	480 nm
$[Na^+]$ = 0 mM	0.16	0.66	0.14	0.62
$[Na^+]$ = 100 mM	0.18	0.66	0.15	0.62

Supplementary Figures

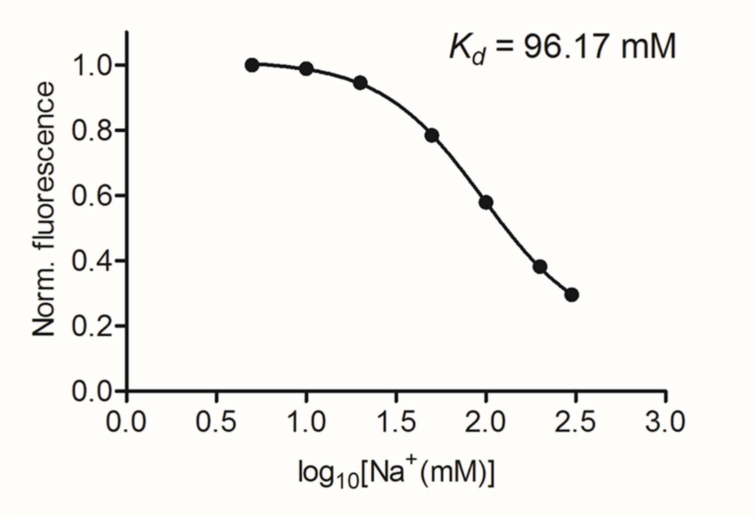

Figure S1. Fluorescence intensity of HaloGFP-Na0.5 as a function of Na^+ concentration. Fluorescence intensity (500 nm excitation; 555 nm emission) of 1 μM HaloGFP-Na0.5 + 2 μM **BCE-HTL$_2$** complex was measured in 30 mM Tris-HCl (pH 7.2) in the absence of KCl. The error bar shows ± S.D. (N = 3)

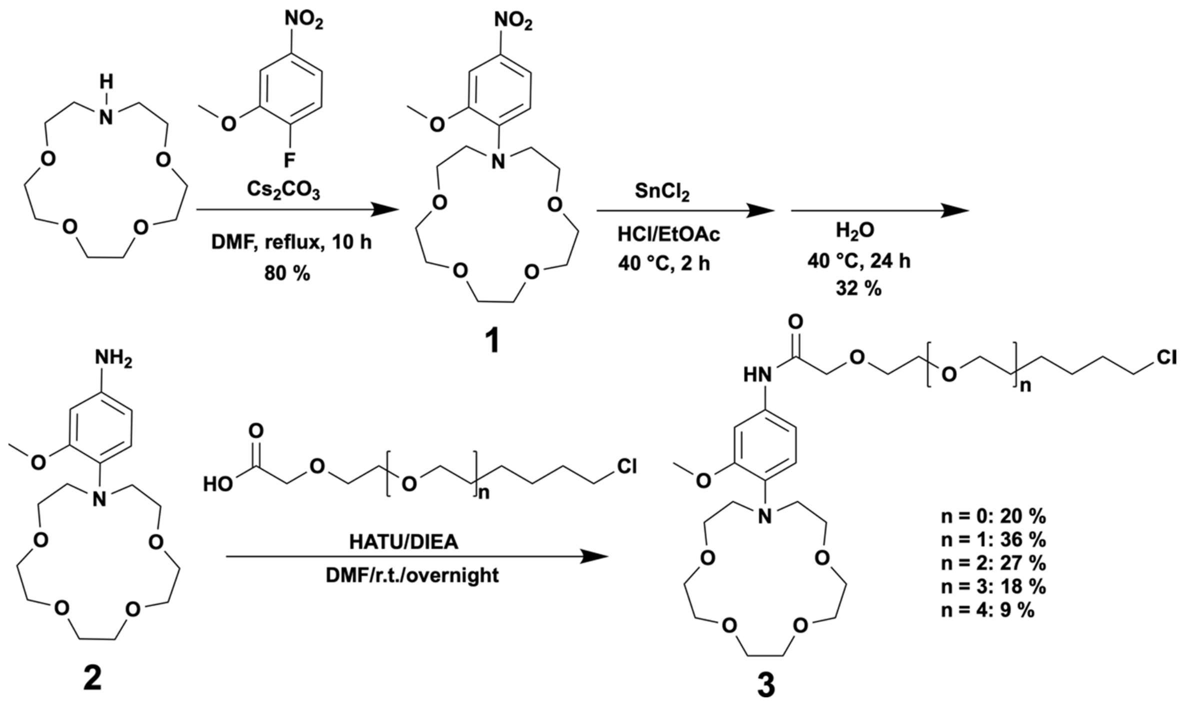

Figure S2. The synthetic scheme used to prepare **ACE-HTL$_{\mathbf{n}}$** (compound **3**, n = 0 - 4).

Compounds **1** and **2**, and the chloroalkane linkers, were synthesized as previously reported.[1,2]

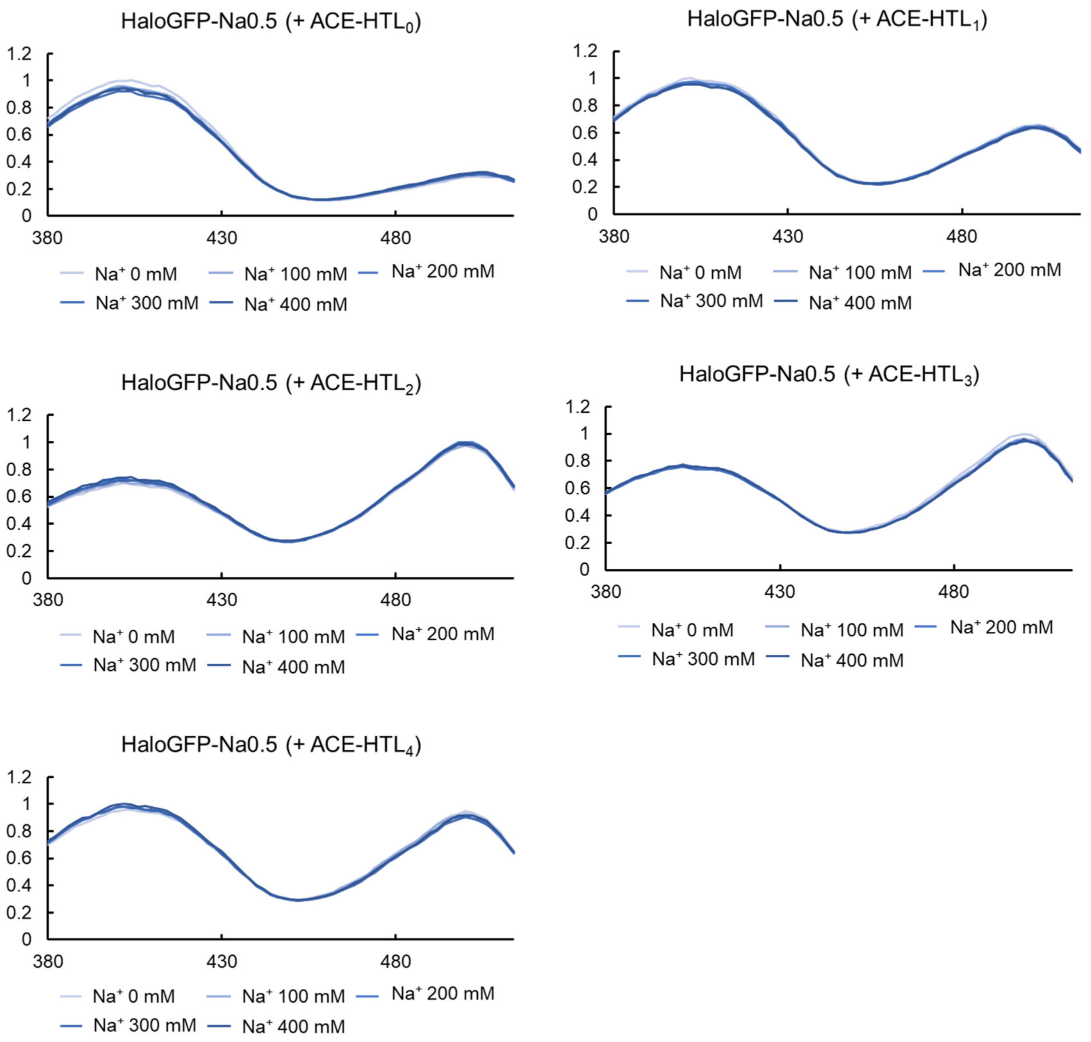

Figure S3. Fluorescence excitation spectra of HaloGFP-Na0.5 + **ACE-HTL$_n$** (n = 0 - 4). Emission wavelength was 555 nm. Measurement was done in 30 mM Tris-HCl (pH 7.2), 0 - 400 mM NaCl, maintaining the ionic strength at 400 mM by addition of KCl.

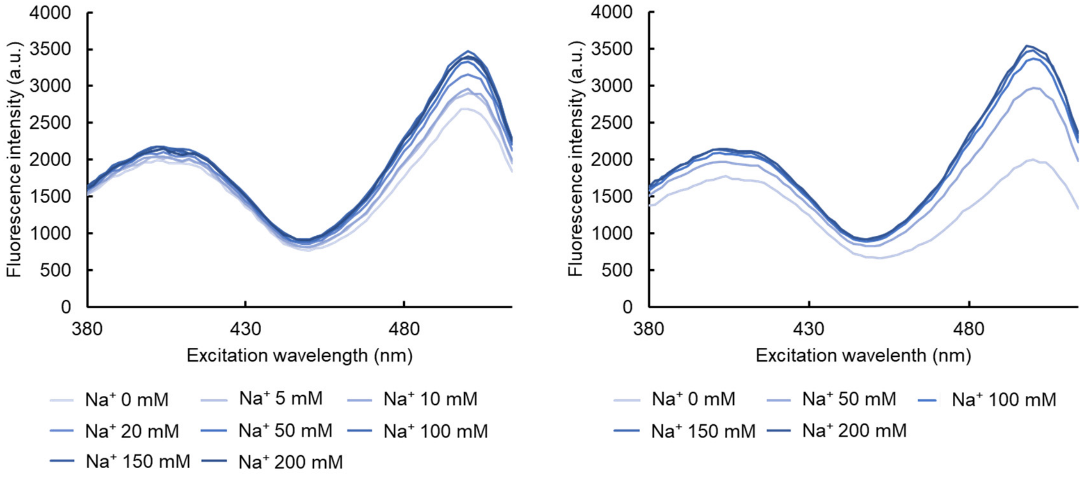

Figure S4. Fluorescence excitation spectra of HaloGFP-Na2.0 + **ACE-HTL$_1$** in the presence (left) and absence (right) of 100 mM KCl. Emission wavelength was 555 nm. Measurement was done in 30 mM Tris-HCl (pH 7.2), with 0 - 200 mM NaCl, and 0 or 100 mM KCl.

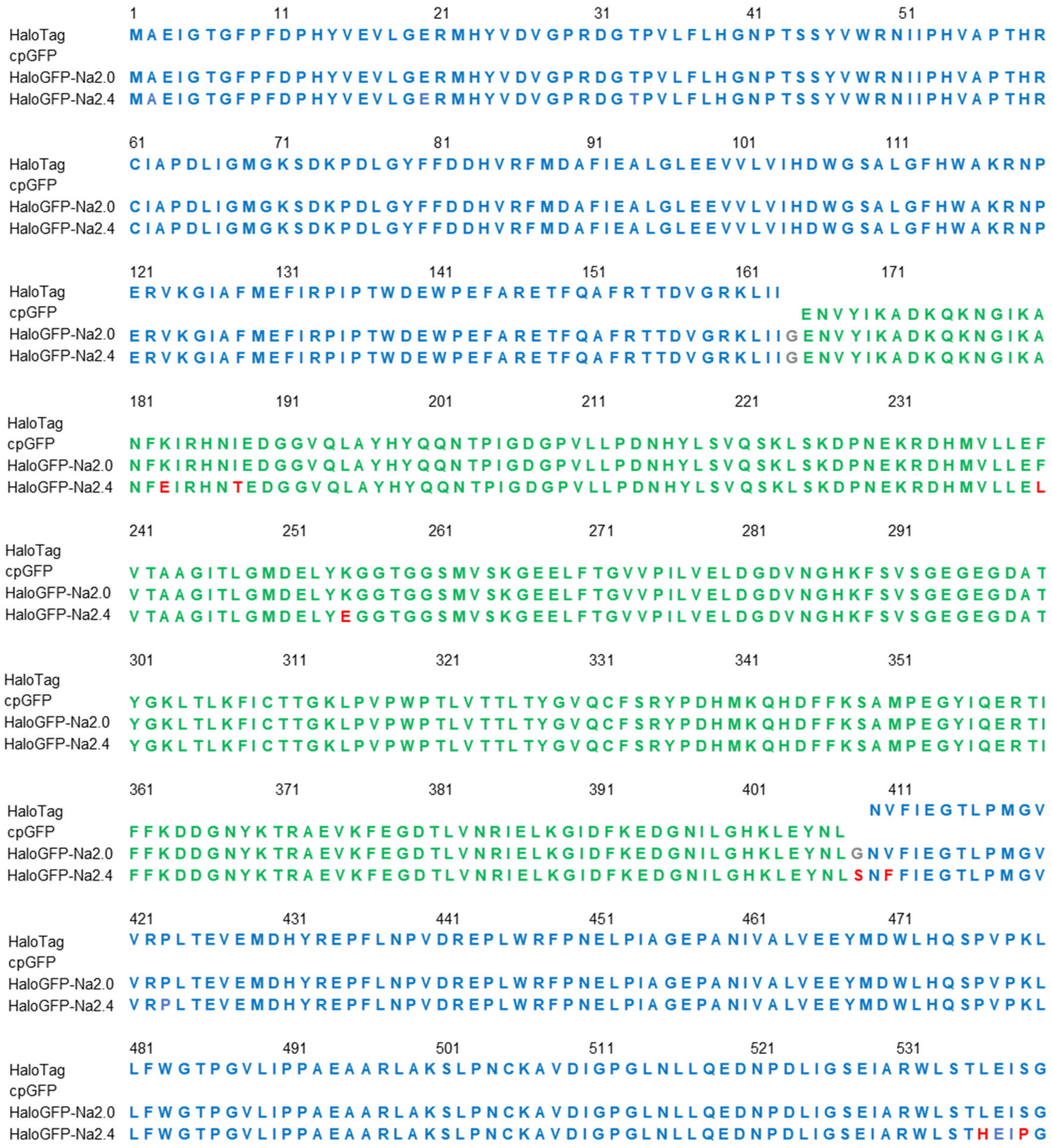

Figure S5. Sequence of HaloGFP-Na2.0 and HaloGFP-Na2.4. The cpGFP is colored in green and the HaloTag protein is colored in blue. Introduced mutations are colored in red.

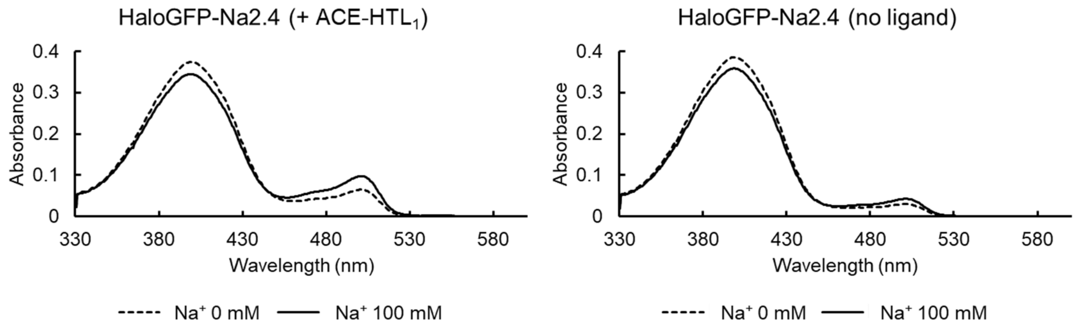

Figure S6. Absorbance spectra of HaloGFP-Na2.4. The spectra were measured with and without **ACE-HTL$_1$** in two buffer conditions. The 0 mM Na^+ buffer contained no salt and the Na^+ 100 mM buffer contained 100 mM NaCl in 30 mM Tris-HCl (pH 7.2). The concentration of the protein was 10 μM and that of **ACE-HTL$_1$** was 20 μM (+ **ACE-HTL$_1$** condition) or 0 μM (no ligand condition).

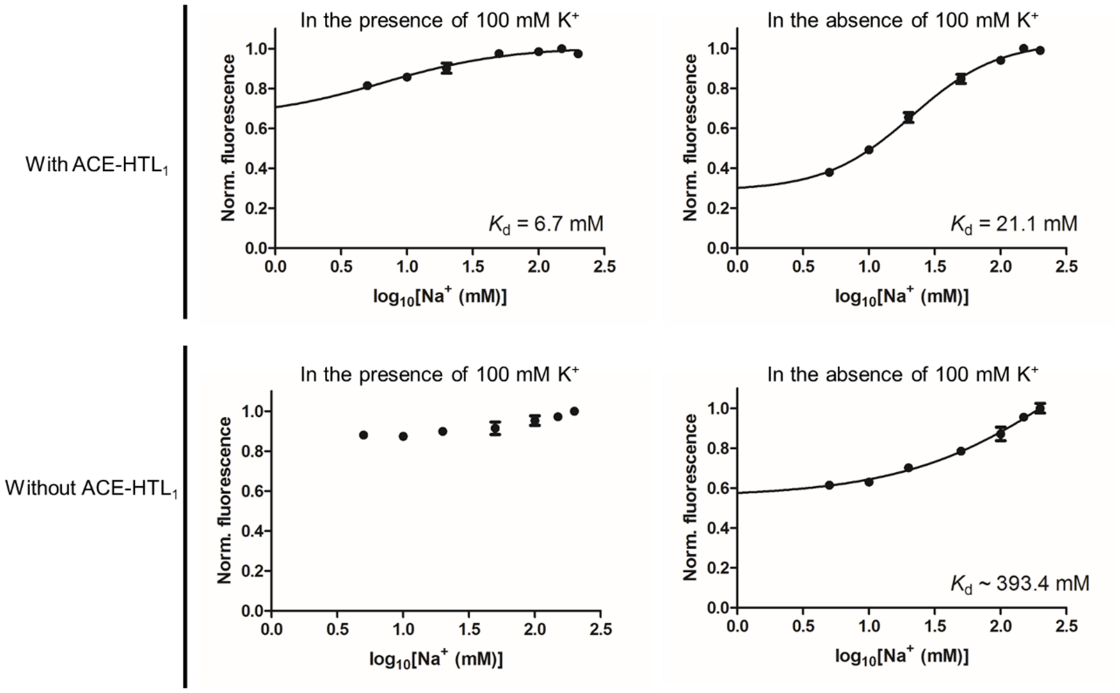

Figure S7. Fluorescence intensity of HaloGFP-Na2.4 as a function of Na^+ concentration. Fluorescence intensity of 1 μM HaloGFP-Na2.4 + 2 μM **ACE-HTL$_1$** complex, and that of 1 μM HaloGFP-Na2.4 only were measured in 30 mM Tris-HCl (pH 7.2) both in the presence of 100 mM KCl (left) and in the absence of 100 mM KCl (right) at 500 nm excitation wavelength and 555 nm emission wavelength. The error bar shows ± S.D. (N = 3)

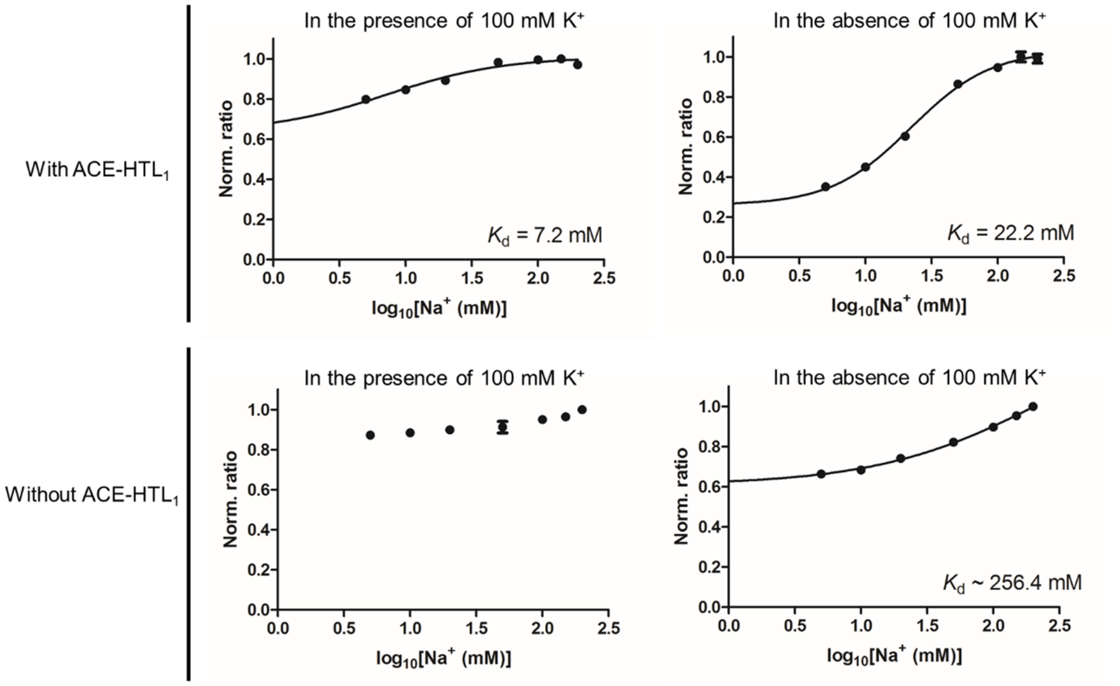

Figure S8. Ratiometric values of HaloGFP-Na2.4 as a function of Na^+ concentration. Ratiometric values of 1 μM HaloGFP-Na2.4 + 2 μM **ACE-HTL$_1$** complex and 1 μM HaloGFP-Na2.4 protein only were measured in 30 mM Tris-HCl (pH 7.2) both in the presence of 100 mM KCl (left) and in the absence of 100 mM KCl (right) at 400 nm and 500 nm excitation wavelength and 555 nm emission wavelength. The error bar shows ± S.D. (N = 3)

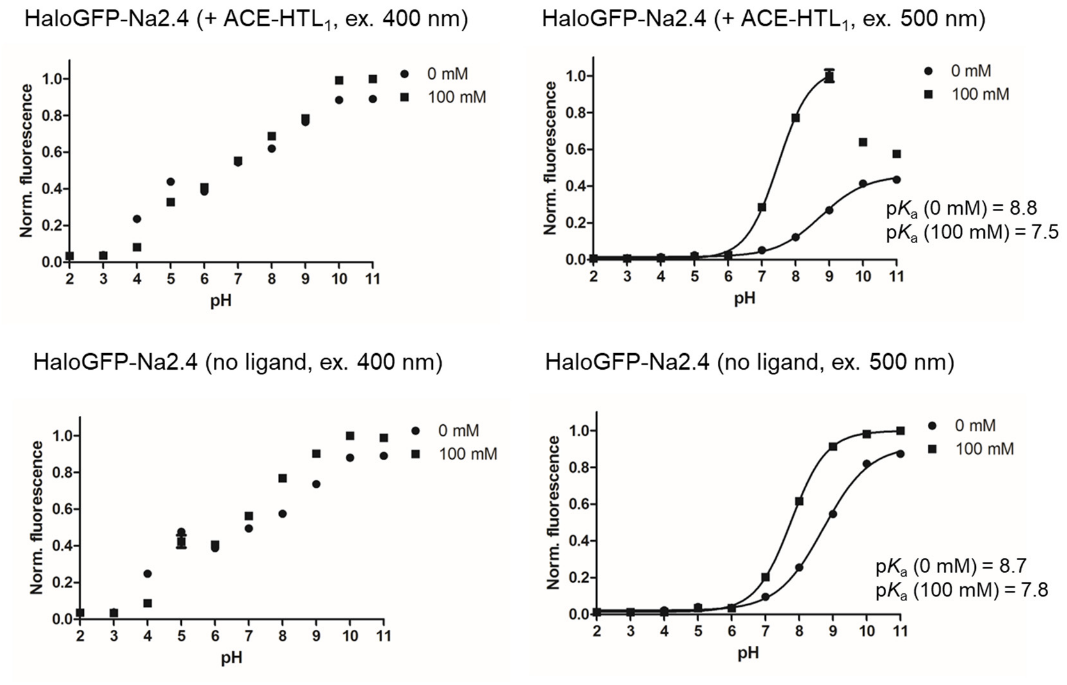

Figure S9. Normalized fluorescence intensity of HaloGFP-Na2.4, either with or without **ACE-HTL$_1$**, at different pH values. Fluorescence intensity of 1 μM HaloGFP-Na2.4 alone or 1 μM HaloGFP-Na2.4 + 2 μM **ACE-HTL$_1$** complex was measured in 1 mM tris (hydroxymethyl)aminomethane/1 mM trisodium citrate dihydrate/1 mM sodium tetraborate decahydrate at 400 nm and 500 nm excitation wavelength and 555 nm emission wavelength. The 0 mM Na^+ buffer contained 0 mM NaCl and the Na^+ 100 mM buffer contained 100 mM NaCl. The error bar shows ± S.D. (N = 3).

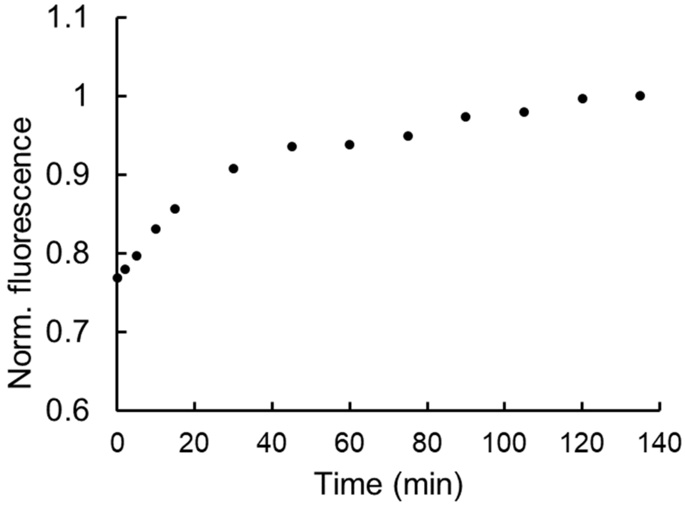

Figure S10. Normalized fluorescence intensity of HaloGFP-Na2.4 + **ACE-HTL$_1$** at the indicated incubation times. Fluorescence intensity of 1 μM HaloGFP-Na2.4 + 1 μM **ACE-HTL$_1$** complex was measured in 30 mM Tris-HCl at pH 7.2 at 500 nm excitation wavelength and 515 nm emission wavelength.

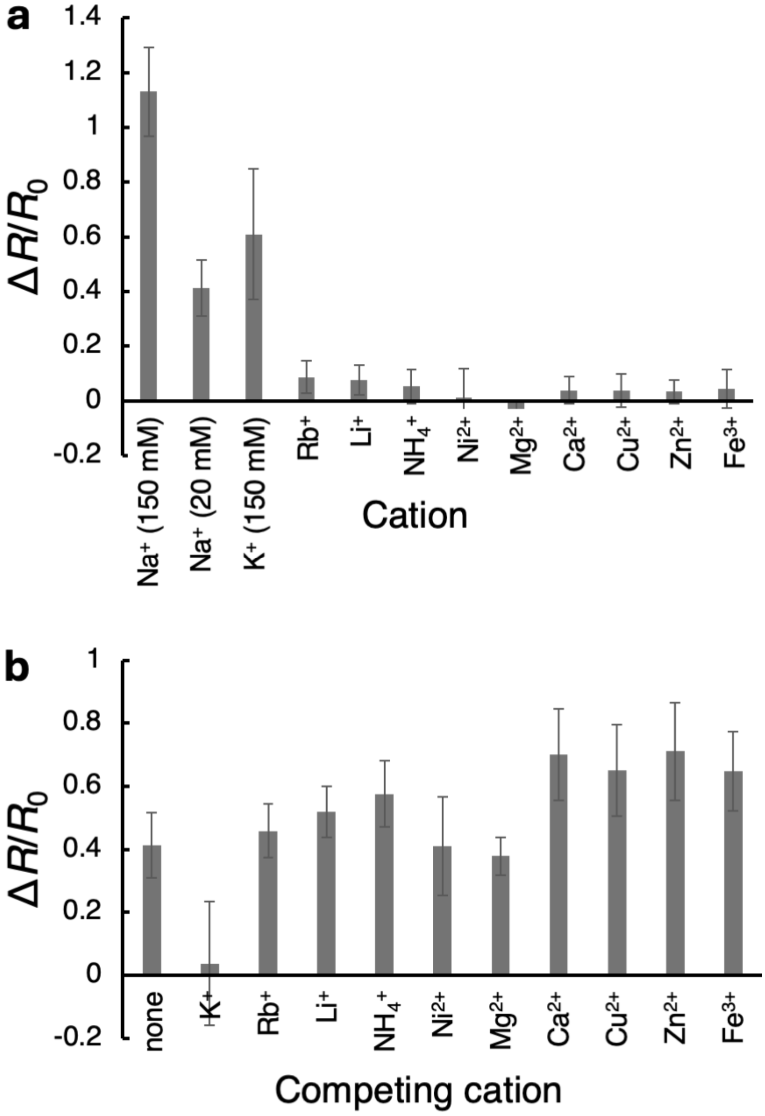

Figure S11. Selectivity of HaloGFP-Na2.4 with **ACE-HTL$_1$** for Na^+ compared to other cations. (a) The changes in excitation ratio of HaloGFP-Na2.4 with **ACE-HTL$_1$** (0.50 μM) in response to various cations were measured in 30 mM Tris-HCl buffer (pH 7.2). [Na^+] (150 mM) = 150 mM, [Na^+] (20 mM) = 20 mM, [K^+] (150 mM) = 150 mM, [Rb^+] = [Li^+] = [NH_4^+] = [Ni^{2+}] = [Mg^{2+}] = 1 mM, [Ca^{2+}] = [Cu^{2+}] = 1 μM, [Zn^{2+}] = 10 nM, and [Fe^{3+}] = 10 μM. The error bar shows ± S.D (N = 3). (b) The changes in excitation ratio of HaloGFP-Na2.4 with **ACE-HTL$_1$** (0.50 μM) in response to 20 mM Na^+ were measured in the presence of various cations (same concentrations as in (a)) in 30 mM Tris-HCl (pH 7.2). The error bar shows ± S.D (N = 3).

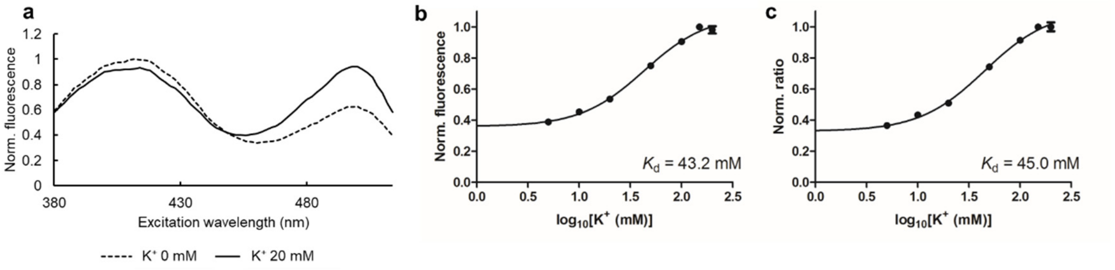

Figure S12. (a) Fluorescence excitation spectra of HaloGFP-Na2.4 for K^+. The spectra were measured for HaloGFP-Na2.4 + **ACE-HTL$_1$** at two buffer conditions (0 mM KCl or 20 mM KCl in 30 mM Tris-HCl (pH 7.2)) at 555 nm emission wavelength. The concentration of HaloGFP-Na2.4 was 1 μM and that of **ACE-HTL$_1$** was 2 μM. (b) Fluorescence intensity of HaloGFP-Na2.4 as a function of K^+ concentration. The fluorescence intensity of 1 μM HaloGFP-Na2.4- 2 μM **ACE-HTL$_1$** complex was measured in 30 mM Tris-HCl (pH 7.2) at 500 nm excitation wavelength and 555 nm emission wavelength. The error bar shows ± S.D. (N = 3). (c) Ratiometric values of HaloGFP-Na2.4 as a function of K^+ concentration. The fluorescence intensity of 1 μM HaloGFP-Na2.4 + 2 μM **ACE-HTL$_1$** complex was measured in 30 mM Tris-HCl (pH 7.2) at 400 nm and 500 nm excitation wavelength and 555 nm emission wavelength. The error bar shows ± S.D. (N = 3).

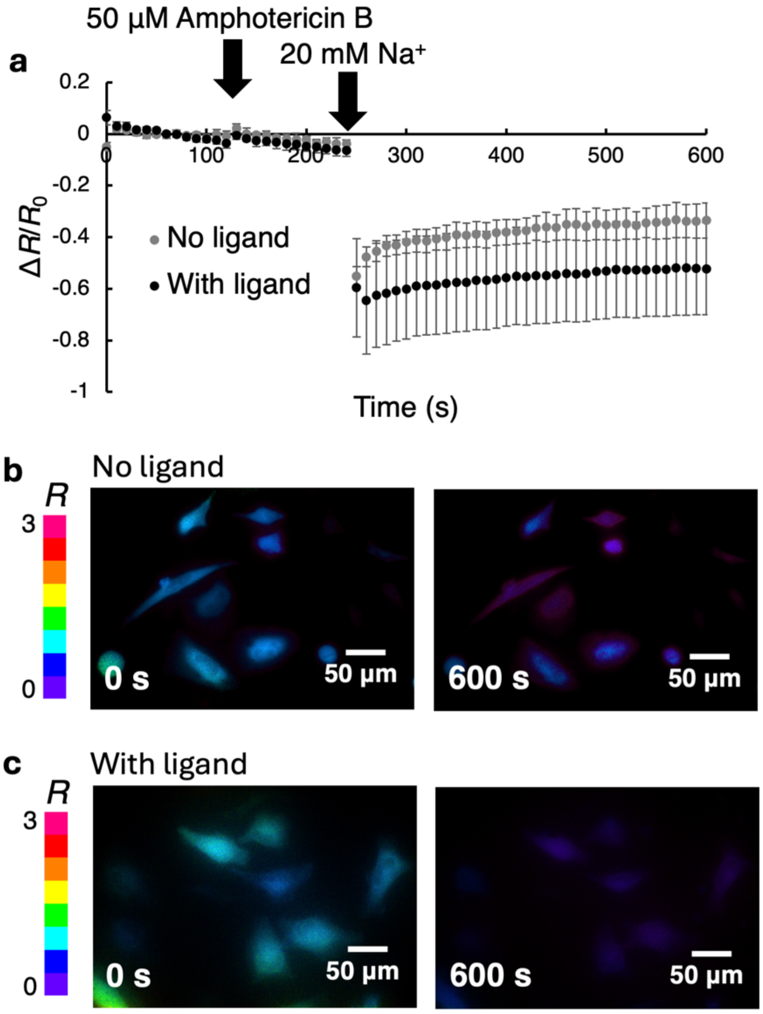

Figure S13. (a) Ratiometric responses to Na^+ treatment for permeabilized HeLa cells expressing the previously reported HaloGFP-Na0.5 and incubated with or without the **BCE-HTL$_2$** ligand. The error bar shows ± S.D. (N = 9 with ligand, N = 11 without ligand) (b,c) Ratiometric images of permeabilized HeLa cells used to acquire the data shown in (a). Shown in (b) are cells that are expressing HaloGFP-Na0.5, but have not been treated with **BCE-HTL$_2$**, before (left image) and after (right image) Na^+ addition. Shown in (c) are cells that that are expressing HaloGFP-Na0.5 and have been treated with **BCE-HTL$_2$**, and have otherwise treated identically to the cells in (b). The color bar shows the color representations for ratios ranging from 0 to 3.

Experimental Methods

Molecular Biology Methods and Instrumentation

Unless otherwise noted, chemicals were supplied by Tokyo Chemical Industries, Aldrich Chemical Co, FUJIFILM Wako Pure Chemical Corporation, Nacalai Tesque, Inc., Thermo Fisher Scientific K.K., Thermo Scientific Invitrogen™, New England Biolabs™, Agilent Technologies™, G-BIOSCIENCES, Calbiochem, Invitrogen, and Promega. They were at the highest grade and were used without further purification.

All NMR were measured with a JEOL JNM-ECS400 at 400 MHz for ^{1}H NMR and 100 MHz for ^{13}C NMR. Mass spectra (MS) were measured with a Bruker Compact System. Column chromatography was conducted with an Isolera-1SW (Biotage) and Biotage Sfär Silica HC D column. HPLC was done with an Inertsil ODS-3 (10.0 × 250 mm) column (GL Sciences Inc.) using an HPLC system composed of a pump (PU-4180, JASCO) and a detector (MD-4015, JASCO).

Phusion high-fidelity DNA polymerase (Thermo Fisher Scientific) was used for PCR amplification. The QuickChange mutagenesis kit (Agilent Technologies) was used for multiple site-directed mutagenesis. Restriction endonucleases, rapid DNA ligation kits and GeneJET miniprep kits were purchased from Thermo Fisher Scientific. PCR products and products of restriction digests were purified using agarose gel electrophoresis (1% agarose, 100 V, 15 min) and the GeneJET gel extraction kit (Thermo Fisher Scientific). Bacterial transformation was performed using electroporation (Bio-rad, 1652100). Protein samples were expressed in *E. coli* strain DH10B (Thermo Fisher Scientific) in LB media with 100 μg/mL ampicillin and 0.02% L-arabinose. Proteins were extracted using B-PER bacterial protein extraction reagent (Thermo Fisher Scientific) according to the manufacturer's instructions. For confirmational assay, the extracted protein was purified with Ni-NTA (Nickel Chelating Resin, G-BIOSCIECES), then buffer exchange was performed to remove imidazole in the elusion buffer. DNA sequence was

read with Sanger sequencing performed by Fasmac Co., Ltd. Absorbance spectra were measured with a Shimadzu UV1800 spectrometer and emission spectra with a JASCO Spectrofluorometer FP-8500. Quantum yield was recorded on a Hamamatsu Photonics C9920-02G. Fluorescence intensity was recorded on a Spark platereader (Tecan). Cell imaging was performed using a IX83 wide-field fluorescence microscopy (Olympus) equipped with a pE-300 LED light source (CoolLED), a 20× (without oil) and 40× objective lens (with oil), an ImagEM X2 EM-CCD camera (Hamamatsu), Cellsens software (Olympus) and a STR stage incubator (Tokai Hit).

Screening of 5 synthetic ligands with 32 HaloGFP protein variants

The 32 HaloGFP variants prepared in our previous work[3] were used for the screening. The proteins were expressed as described above. After nickel purification and buffer exchange, the concentration of the protein liquid was measured by DeNovix™ Spectrophotometer. For the measurement with ligand, 250 μM **ACE-HTL$_n$** (in DMSO, n = 0~4, 5 kinds for each protein) were added so that the molar concentration of protein and chelator was at a ratio of 1:2. As for the measurement without **ACE-HTL$_n$**, just DMSO was added with the same amount in the case of with ligand condition. Then the sample (both with and without **ACE-HTL$_n$**) was shaken on ice for 90 minutes. 10 μL of the sample and 90 μL of the buffer were placed into 96 well plate (Thermo Fisher Scientific™ Nunc MicroWell 96-Well Optical Bottom Plates). The sample was measured in the five buffer conditions (30 mM Tris, 100 mM KCl, 0/25/50/100/200 mM NaCl, pH 7.2). The plate was measured by fluorescence spectrometer (TECAN, Spark®) with excitation spectra mode from 380 nm to 515 nm.

Library preparation for directed evolution

DNA libraries for directed evolution were prepared by two methods.

1. Random Mutagenesis

Using error prone PCR, mutations were randomly introduced in the DNA encoding the whole gene of HaloGFP-Na (from N-terminal of HaloTag to C-terminal of HaloTag) at Round 1, 2, and 4. Forward and reverse primers were designed to cover from the restriction enzymes outside of HaloTag to the start or stop codon, to not alter the start and stop codons of HaloGFP-Na, and to introduce mutations in the full range of HaloGFP-Na (shown in the figure below). Mn^{2+} concentration was controlled so that the error rate should be about three amino acid mutations in the full range of HaloGFP-Na on average. The plasmid library was constructed by restriction enzyme-ligation by introducing the PCR products into a pBAD vector.

2. Linker Optimization

Libraries were constructed by linker optimization by introducing mutagenesis only in the linker region at Round 3, using QuickChange Lightning Multi Site-Directed Mutagenesis Kit. The linker here refers to the amino acids that connect HaloTag to EGFP (shown in the figure below). Primers were designed so that they already contained the desired mutations. Plasmid was constructed in the standard PCR condition with the primers and nicks were connected by the blended enzymes of the kit. Two primers were designed for introducing mutagenesis into linker 1 (162nd - 165th amino acids) and linker 2 (407th - 410th amino acids) by replacing these four amino acids with NNK (N indicates A, T, G, or C, and K indicates T or G). To degrade the template plasmid, the PCR product was treated with DpnI.

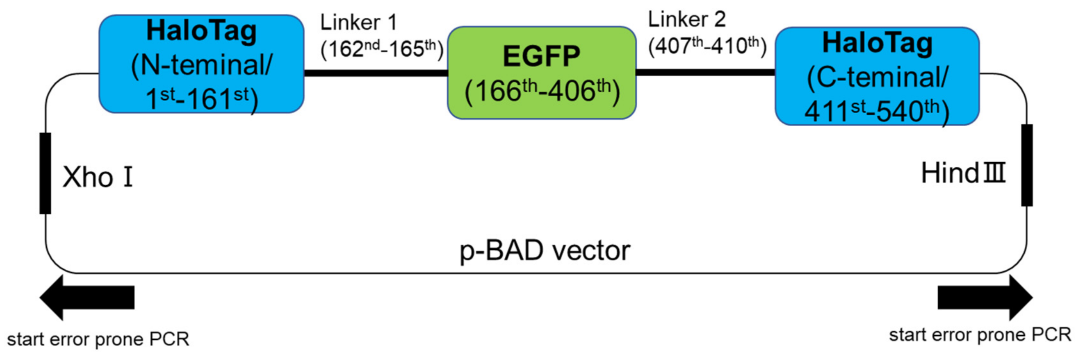

Figure: Schematic structure of HaloGFP-Na plasmid.

Fluorescent assay for directed evolution

Measurement 1: 96-deep well plate-based high-throughput screening

After culturing, the 96 deep well plate was centrifuged for 10 minutes at 4000 rcf and the supernatant was discarded. 50 μL of B-PER was added and vortexed for 30 seconds. The plate was placed on ice and shaken for 30 minutes. Into the protein solution extracted with B-PER, 200 μL of Tris buffer (30 mM Tris (hydroxymethyl) aminomethane, 100 mM KCl, pH 7.2) and 4 μL of **ACE-HTL$_1$** (125 μM) were added and the plate was vortexed for 10 seconds. The plate was shaken on ice for 90 minutes. After that, the plate was centrifuged for 10 minutes at 4000 rcf and 90 μL of the solution was transferred to two 96 well plates. The plates were measured by the fluorescent spectrometer (TECAN, Spark®) with excitation spectra mode from 380 nm to 515 nm. The plate was taken out and 2 μL of Na^+ (+) buffer was added into the plate. The plate was measured again with the same measurement parameter. After that, 8 μL of Na^+ (+) buffer was added into the same plate and measured again with the same measurement parameter. Na^+ (+) buffer: 30 mM Tris (hydroxymethyl) aminomethane, 100 mM KCl, 500 mM NaCl, pH 7.2.

Measurement 2: Detailed fluorescence assay after high-throughput screening

The DNA of selected variants was extracted from the remaining pellet at the bottom of 96 deep well plate. The DNA was transformed into *E. coli* and cultured in arabinose/ampicillin enriched LB. The protein was extracted and treated with Ni-NTA purification and buffer exchange. The concentration of the protein liquid was measured by DeNovix™ Spectrophotometer.

As for the measurement with ligand, 250 μM **ACE-HTL$_1$** in DMSO was added so that the molar concentration of protein and chelator was at a ratio of 1:2. As for the measurement without **ACE-HTL$_1$**, just DMSO was added with the same amount in the case of with **ACE-HTL$_1$** condition. Then the sample (both with and without chelator) was shaken on ice for 90 minutes. 10 μL of the sample and 90 μL of the buffer were placed into 96 well plate (Thermo

Fisher Scientific^{TM} Nunc MicroWell 96-Well Optical Bottom Plates). The sample was measured in the three different series of buffer conditions. Series 1 was 30 mM Tris, 100 mM KCl, 0/5/10/20/50/100/150/200 mM NaCl, pH 7.2. Series 2 was 30 mM Tris, 0/25/50/100/150 mM NaCl, pH 7.2. Series 3 was 30 mM Tris, 0/25/50/100/150 mM KCl, pH 7.2. The plate was measured by fluorescent spectrometer (TECAN, Spark®) with excitation spectra mode from 380 nm to 515 nm.

K_d determination

In the screening and directed evolution, the analysis of each variant's approximate K_d was done with the value $\Delta F/F_0$ at 500 nm excitation wavelength for the different concentration of Na^+ with an online EC50 calculator (https://www.aatbio.com/tools/ec50-calculator).

For more precise K_d determination, the purified protein solution was diluted into a series of buffers. The fluorescence intensity or ratio of the indicator in each solution was measured and subsequently plotted as a function of Na^+ concentration. The data were fitted to the Hill equation to obtain K_d and the apparent Hill coefficient. The plotted curve was detected using Graph Pad Prism 5 with the formula "log(agonist) vs. response — Variable slope (four parameters)".

Characterization *in vitro*

The protein was extracted and treated with Ni-NTA purification and buffer exchange. The concentration of the protein liquid was measured by DeNovix^{TM} Spectrophotometer.

As for the measurement with **ACE-HTL$_1$**, **ACE-HTL$_1$** in DMSO was added so that the molar concentration of protein and **ACE-HTL$_1$** was at a ratio of 1:2 (except for labelling kinetics). As for the measurement without **ACE-HTL$_1$**, just DMSO was added with the same amount in the case of with ligand condition. Then the sample (both with and without **ACE-HTL$_1$**) was shaken on ice for 90 minutes. After that, the sample was mixed with the buffers and

used for each measurement (absorbance, emission, excitation, pK_a, K_d, quantum yield, selectivity and labelling kinetics).

Cell culture

The type of cells was HeLa cells (American Type Culture Collection (ATCC)). The gene encoding HaloGFP-Na2.4 or HaloGFP-Na0.5 was inserted into the pcDNA3.1 vector between the XhoI and HindIII restriction sites. Cells are cultured in Dulbecco's modified Eagle medium (DMEM; Nakalai Tesque) enriched with 10% fetal bovine serum (FBS; Sigma-Aldrich) and 1% penicillin-streptomycin (Nakalai Tesque) at 37°C and 5% CO_2. Cells were passaged while they were in log-phase of their growth. Cells (~2×10^5) were seeded into 35-mm glass-bottom cell-culture dishes (Iwaki) with 200 μL opti-MEM (Thermo Fisher Scientific) and transfected with the plasmid (2 μg) using 4 μL polyethyleneimine (Polysciences, 1 mg polyethyleneimine of was diluted in 1 mL ultrapure water). DMEM was exchanged with fresh DMEM after 4 hours from transfection. After overnight incubation, the transfected cells were replaced onto 10-mm glass-bottom dishes (Iwaki) for the cell imaging.

Cell imaging of HaloGFP-Na derivatives

Transfected cells were imaged between 48-72 hours after transfection. The filter sets for imaging of HaloGFP-Na2.4 and HaloGFP-Na0.5 were: excitation 405/20 nm, dichroic mirror 425-nm dclp, and emission 518/45 nm; excitation 470/20 nm, dichroic mirror 490-nm dclp, and emission 518/45 nm.

For imaging of HaloGFP-Na2.4, HeLa cells transfected with HaloGFP-Na2.4 were incubated with **ACE-HTL$_1$** in DMEM (1 μM) for 90 minutes at 37°C. Then, the dish (Iwaki, 10 mm glass bottom) was washed twice with the imaging buffer, and the buffer was exchanged to 1800 μL of the imaging buffer just before the imaging. At indicated time, amphotericin B (Tokyo Chemical Industries, 50 μM final concentration), NaCl aq. (20 mM final concentration)

were added. The imaging buffer was 10 mM HEPES with 145 mM *N*-methyl-D-glucamine (pH 7.2). For imaging of HaloGFP-Na0.5, the same procedure was conducted using $\mathbf{BCE\text{-}HTL_2}$.

Synthetic Methods

Synthesis of Compound 1

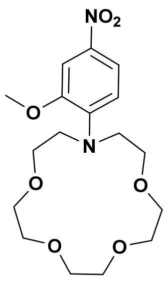

1

A mixture of 1-aza-15-crown-5 (841.1 mg, 3.84mmol), the corresponding 1-fluoro-2-methoxy-4-nitrobenzene (3.27 g, 19.12 mmol) and Cs_2CO_3 (1.24 g, 3.82 mmol) in DMF (9 mL) was refluxed for 10 h. After cooling down to room temperature the residue was filtered off and the organic phase was concentrated in vacuo. The resulting residue was purified by column chromatography on silica using $CHCl_3$ /CH_3OH (v/v, 99/1 to 95/5) as eluent mixtures to afford Compound **1** as a yellow (brown) oil in 80 % yield.

^{1}H NMR ($CDCl_3$, 400 MHz) δ 7.82 (dd, J = 8.9, 2.5 Hz, 1H), 7.66 (d, J = 2.3 Hz, 1H), 6.90 (d, J = 9.2 Hz, 1H), 3.87 (s, 3H), 3.75-3.72 (m, 4H), 3.67-3.63 (m, 16H); HRMS (m/z): [M+Na$^+$] calcd. for $C_{17}H_{26}N_2O_7$, 393.1632; found, 393.1659

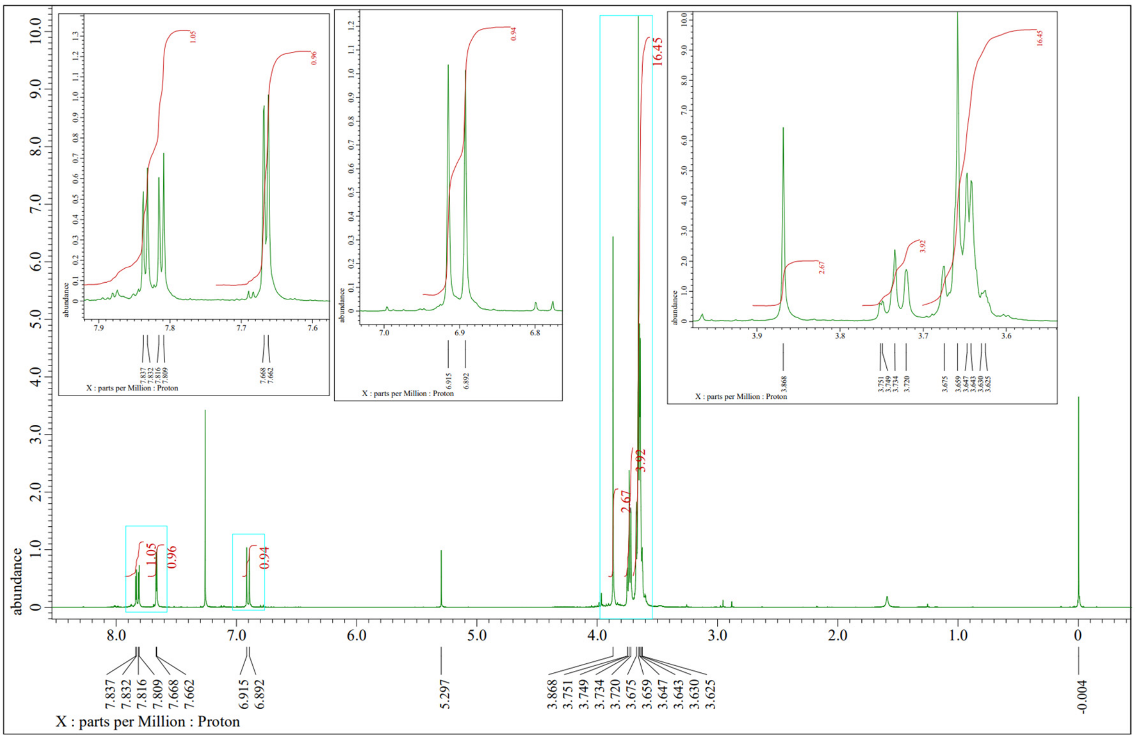

Synthesis of Compound 2

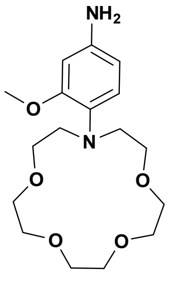

2

The nitro compound **1** (363.0 mg, 0.98 mmol) was added to a solution of $SnCl_2$ (235.8 mg, 1.24 mmol) in conc. HCl (0.62 mL) and ethyl acetate (2.07 mL) and stirred for 2 h at 40 °C. After that, H_2O (2.07 mL) was added and the resulting solution was stirred for 24 h at 40 °C. Afterwards, the solution was adjusted to a pH value of 8 with 1 M NaOH and extracted with CH_2Cl_2 (100 mL), separated, dried with $MgSO_4$ and concentrated in vacuo. The resulting residue was purified by column chromatography on silica with $CHCl_3$ /CH_3OH (v/v, 1-15% CH_3OH) as an eluent mixture to afford Compound **2** as a brownish oil in 32 % yield.

^1H NMR ($CDCl_3$, 400 MHz) δ 6.98 (d, J = 7.8 Hz, 1H), 6.23 (dd, J = 10.3, 2.5 Hz, 2H), 3.78 (s, 3H), 3.70 (s, 4H), 3.68-3.59 (m, 15H), 3.34 (t, J = 6.0 Hz, 4H); HRMS (m/z): [M+Na^+] calcd. for $C_{17}H_{28}N_2O_5$, 363.1890; found, 363.1890

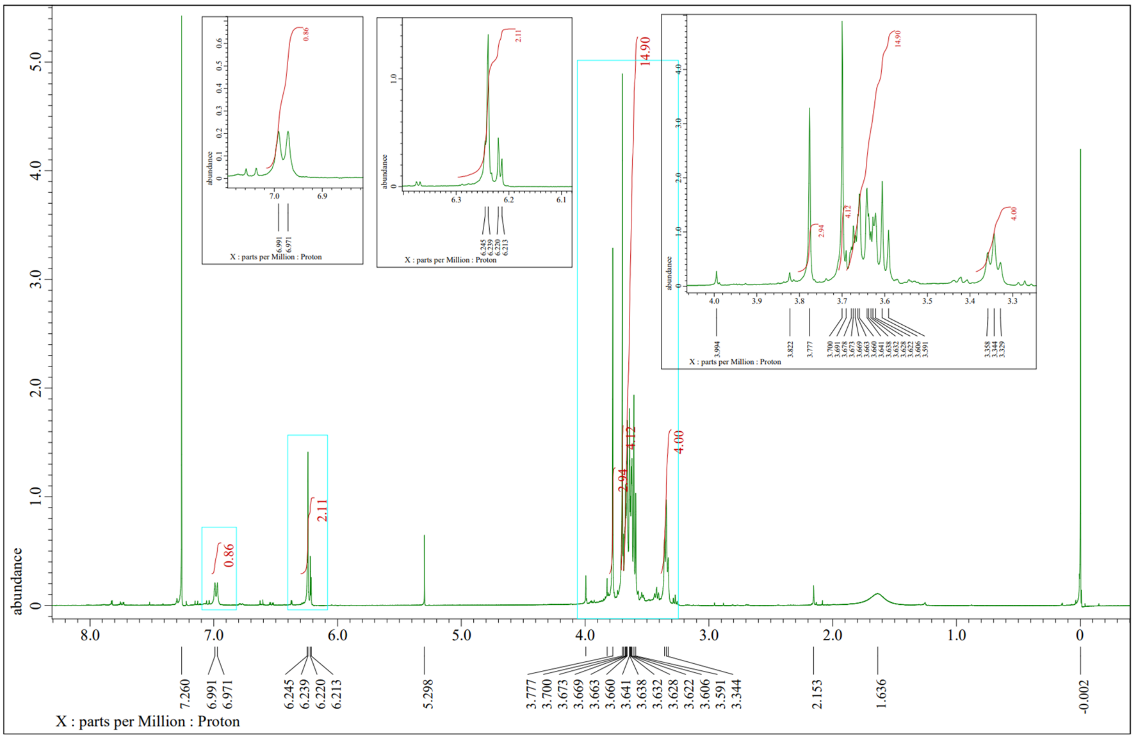

Synthesis of ACE-HTL$_n$ (Compound 3, n = 0-4)

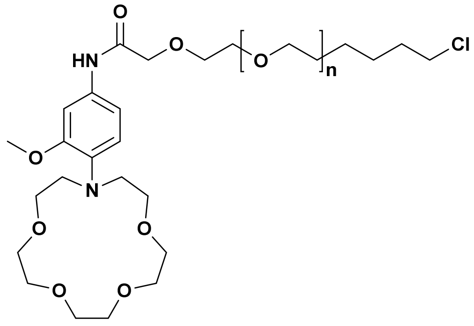

3

Compound **2** (1 eq), chloroalkane linker (1.1 eq, synthesized according to ref. 1), HATU (1.1 eq), DIEA (3 eq), and molecular sieves were added into DMF (1.0 mL). The mixture was stirred at room temperature overnight. Extra linker and HATU were added during the reaction only in the synthesis of Compound **3** (n= 2). After the reaction, the organic solvent was removed with a rotary evaporator. The residue was purified by silica gel chromatography with CH_2Cl_2 /CH_3OH (v/v, 1-20% CH_3OH), then purified by HPLC (solvent A: H_2O/0.1% TFA aq., solvent B: 80% CH_3CN/0.1% TFA aq., A/B = 0-80% (v/v of B)) to give Compound **3**. The quantities of reagents and resulting yields are summarized in the following table.

The amounts of reagents used and the yields

	**n = 0**	**n = 1**	**n = 2**	**n = 3**	**n = 4**
**Compound 2 (mg)**	33.2	40.7	34.1	33.1	26.0
**Linker (mg)**	22.0	33.0	31.0 (+ 29.1)	14.9	46.2
**HATU (mg)**	44.1	52.9	43.8 (+ 42.9)	43.2	43.7
**DIEA (mg)**	50.6	52.0	41.8	43.2	49.7
**Yield (%)**	20	36	27	18	9

ACE-HTL$_0$ (Compound 3, n = 0)

^{1}H NMR (400 MHz, CDCl$_3$) δ 8.64 (s, 1H), 7.73 (t, J = 8.5 Hz, 2H), 7.03 (d, J = 7.3 Hz, 1H), 4.07 (s, 2H), 3.96 (s, 3H), 3.87 (d, J = 2.3 Hz, 4H), 3.70-3.51 (m, 20H), 1.84-1.77 (m, 2H), 1.75-1.61 (m, 2H), 1.54-1.39 (m, 4H); ^{13}C NMR (100 MHz, CDCl$_3$) δ 168.6, 153.0, 139.6, 125.6, 112.3, 104.7, 72.0, 70.6, 70.3, 69.7, 69.6, 65.3, 56.5, 45.1, 32.6, 29.4, 26.7, 25.5; HRMS (m/z): [M+H$^+$] calcd. for $C_{25}H_{41}ClN_2O_7$, 517.2675; found, 517.2710

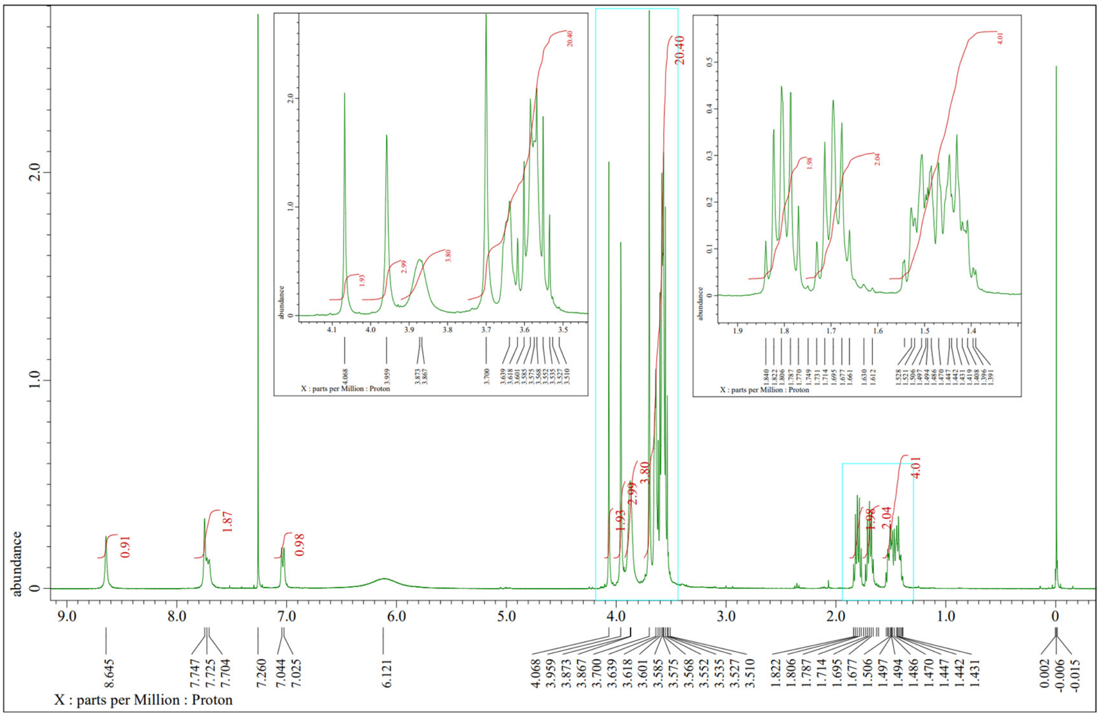

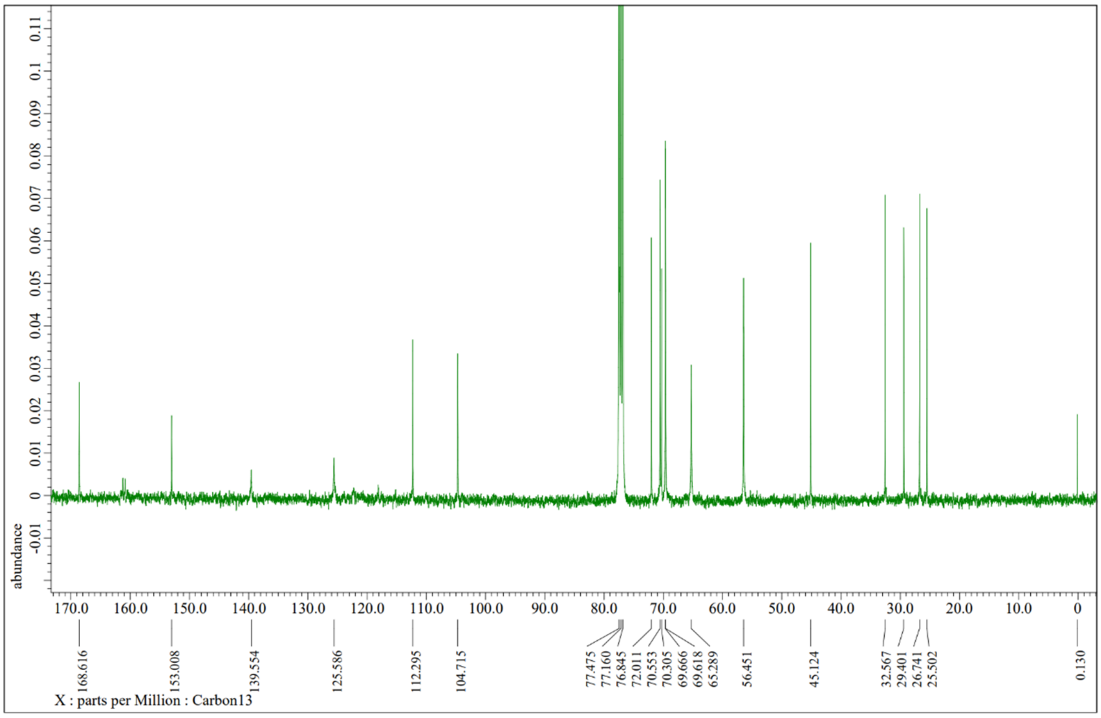

ACE-HTL$_1$ (Compound 3, n = 1)

^{1}H NMR (400 MHz, $CDCl_3$) δ; 9.06 (s, 1H), 7.78 (d, J = 27.5 Hz, 2H), 7.09 (dd, J = 8.7, 1.4 Hz, 1H), 4.14 (s, 2H), 3.98 (s, 7H), 3.78-3.49 (m, 24H), 1.78-1.71 (m, 2H), 1.67-1.60 (m, 2H), 1.48-1.33 (m, 4H); ^{13}C NMR (100 MHz, $CDCl_3$) δ; 169.3, 152.7, 141.5, 126.5, 112.5, 104.8, 71.6, 71.5, 70.5, 70.5, 69.7, 69.2, 69.1, 63.6, 57.2, 56.6, 45.1, 32.6, 29.4, 26.7, 25.4; HRMS (m/z): [M+H$^+$] calcd. for $C_{27}H_{45}ClN_2O_8$, 561.2937; found, 561.2958

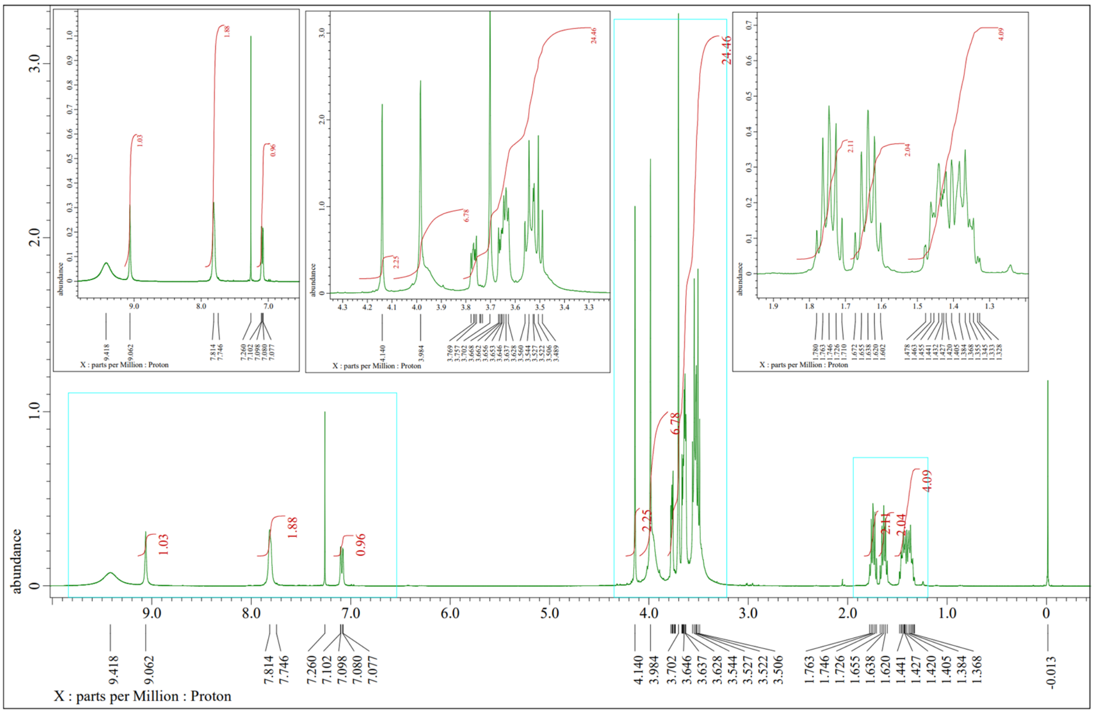

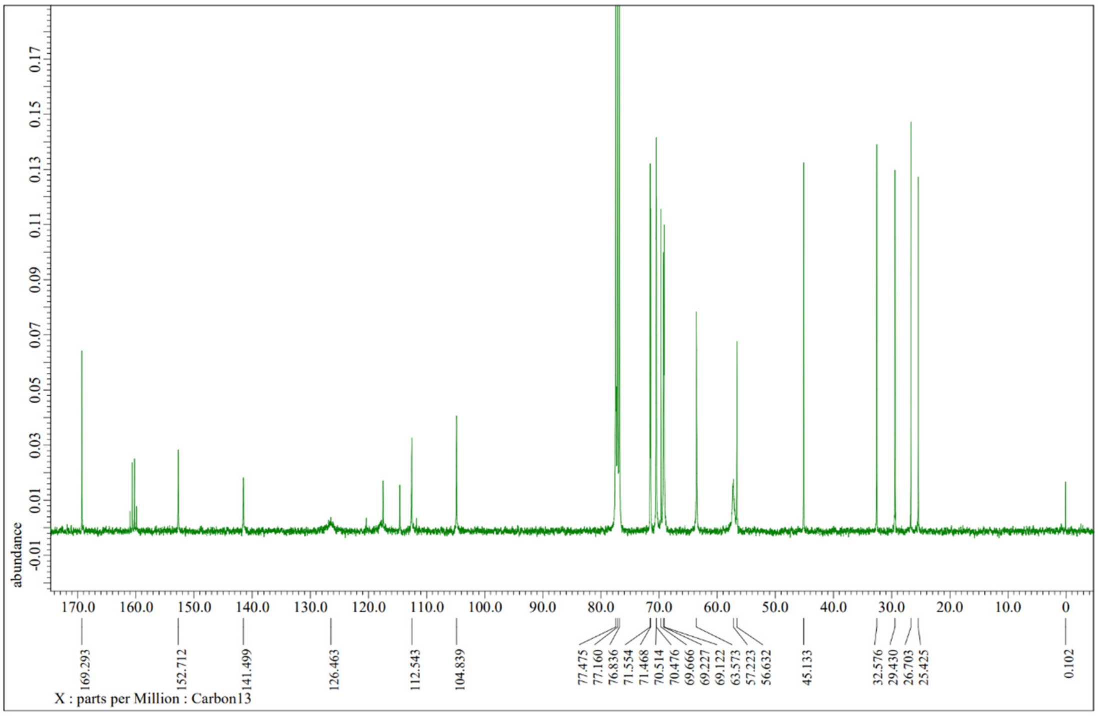

ACE-HTL$_2$ (Compound 3, n = 2)

^{1}H NMR (400 MHz, CDCl$_3$) δ; 9.16 (s, 1H), 7.82-7.78 (m, 2H), 7.14 (dd, J = 8.7, 1.8 Hz, 1H), 4.13 (s, 2H), 3.97 (s, 7H), 3.77-3.43 (m, 28H), 1.77-1.70 (m, 2H), 1.59-1.52 (m, 2H), 1.45-1.29 (m, 4H); ^{13}C NMR (100 MHz, CDCl$_3$) δ; 169.2, 152.8, 140.9, 125.9, 112.6, 104.8, 71.4, 71.3, 70.8, 70.5, 70.1, 69.9, 69.6, 69.5, 64.5, 57.0, 56.6, 45.2, 32.6, 29.5, 26.8, 25.5; HRMS (m/z): [M+H$^+$] calcd. for $C_{29}H_{49}ClN_2O_9$, 605.3199; found, 605.3229

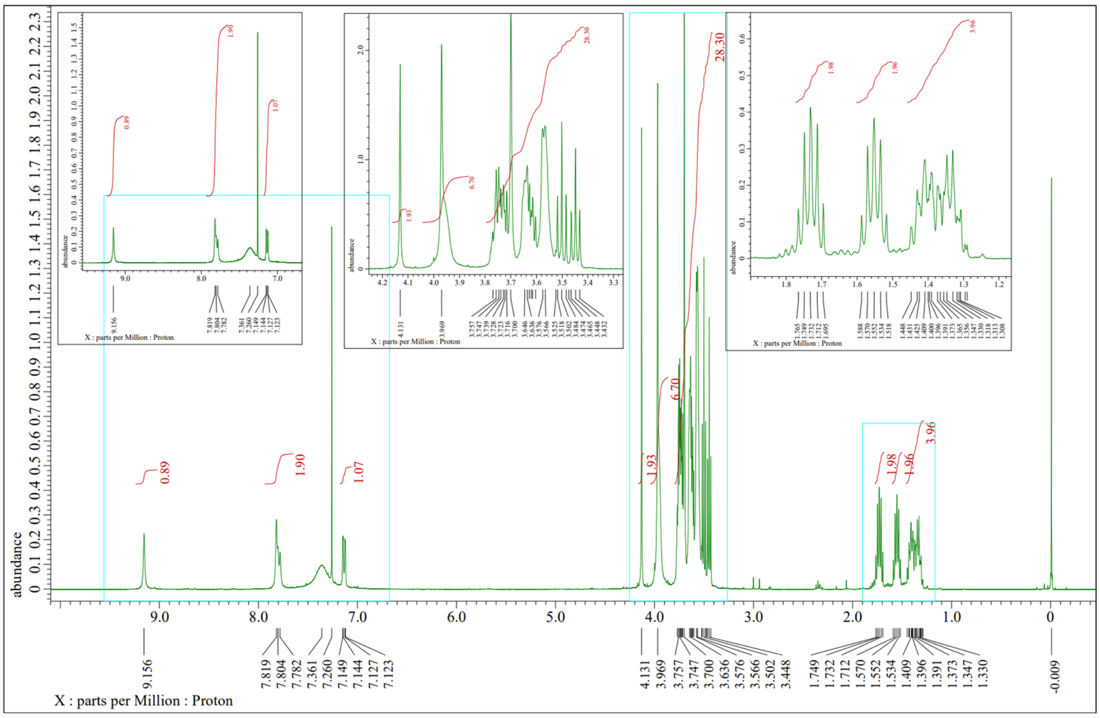

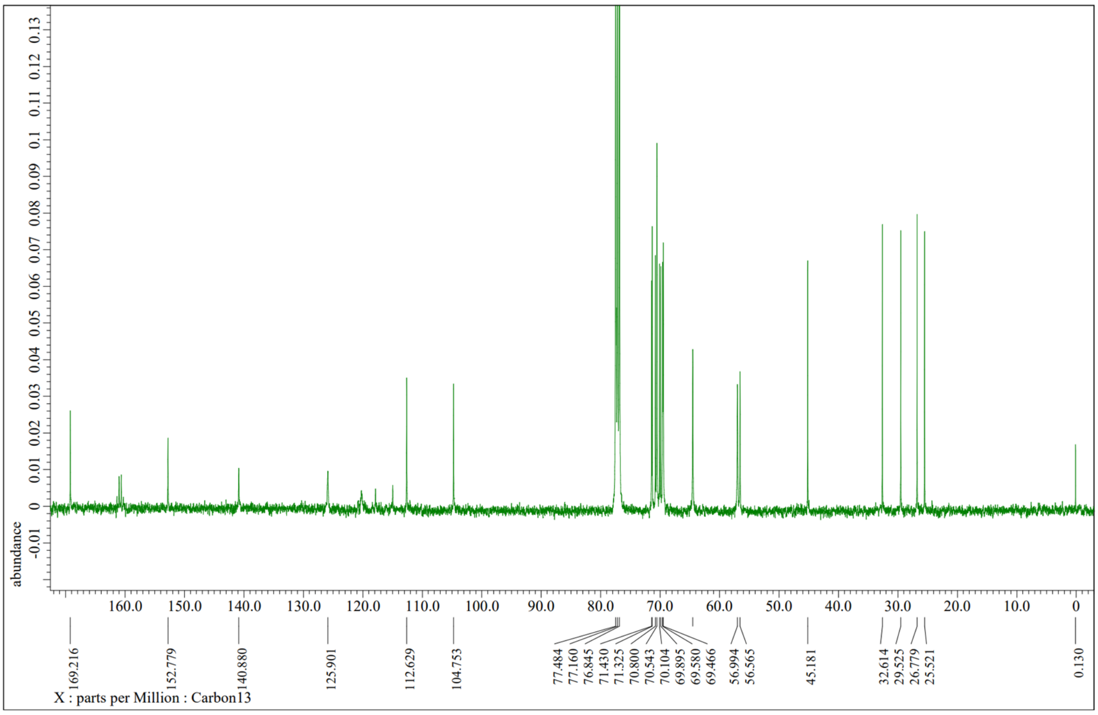

ACE-HTL$_3$ (Compound 3, n = 3)

^{1}H NMR (400 MHz, $CDCl_3$) δ; 8.98 (s, 1H), 7.68 (s, 1H), 7.56 (d, J = 6.4 Hz, 1H), 7.03 (d, J = 6.9 Hz, 1H), 4.12 (s, 2H), 3.92 (s, 3H), 3.82-3.40 (m, 36H), 1.79-1.72 (m, 2H), 1.60-1.53 (m, 2H), 1.47-1.30 (m, 4H); ^{13}C NMR (100 MHz, $CDCl_3$) δ; 168.9, 153.0, 139.0, 124.8, 112.5, 104.8, 71.4, 71.3, 70.8, 70.7, 70.6, 70.5, 70.2, 69.9, 66.1, 56.3, 56.0, 45.2, 32.7, 29.6, 26.8, 25.5; HRMS (m/z): [M+H$^+$] calcd. for $C_{31}H_{53}ClN_2O_{10}$, 649.3462; found, 649.3498

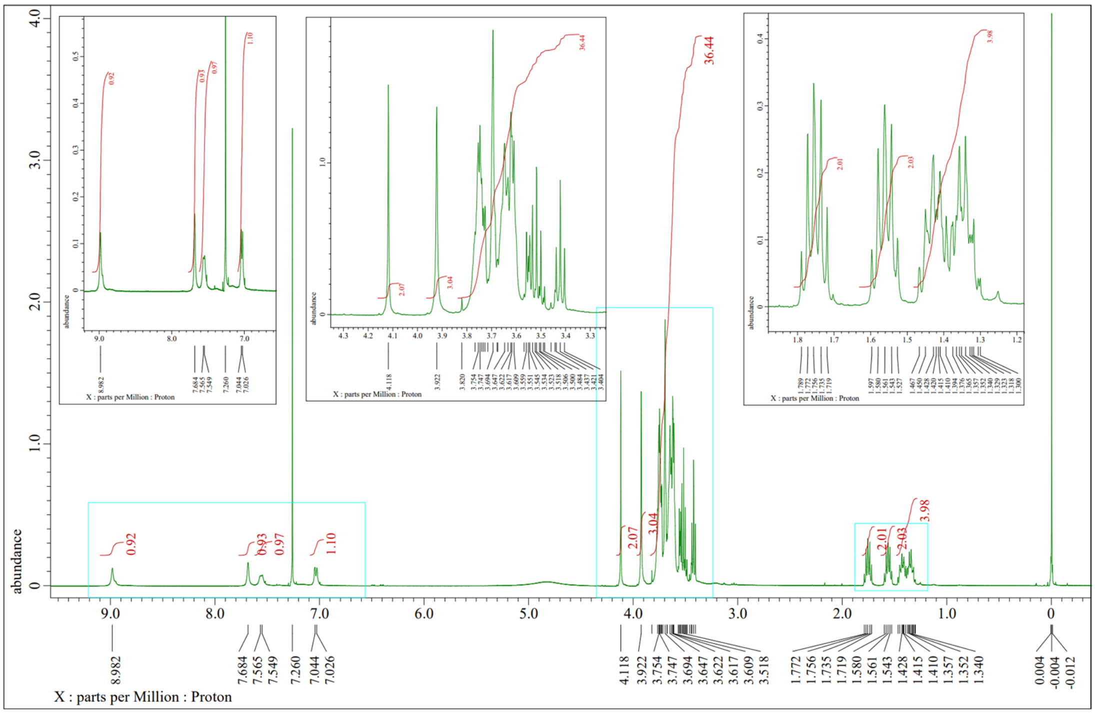

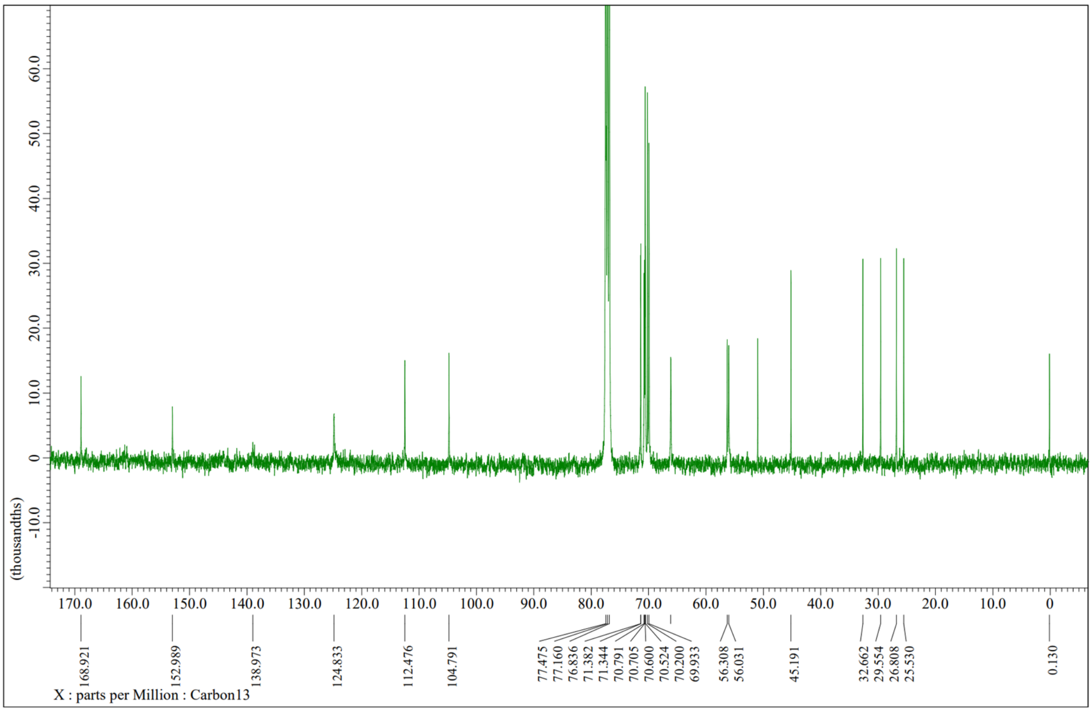

ACE-HTL$_4$ (Compound 3, n = 4)

^{1}H NMR (400 MHz, $CDCl_3$) δ; 9.20 (s, 1H), 7.81-7.75 (m, 2H), 7.14 (dd, J = 8.7, 1.8 Hz, 1H), 4.14 (s, 2H), 3.95 (d, J = 15.1 Hz, 7H), 3.77-3.40 (m, 36H), 1.79-1.72 (m, 2H), 1.61-1.53 (m, 2H), 1.47-1.30 (m, 4H); ^{13}C NMR (100 MHz, $CDCl_3$) δ; 169.2, 152.8, 140.5, 125.6, 112.6, 104.8, 71.4, 70.7, 70.7, 70.6, 70.4, 70.2, 69.7, 69.6, 64.9, 56.8, 56.5, 45.2, 32.7, 29.6, 26.8, 25.5; HRMS (m/z): [M+H$^+$] calcd. for $C_{33}H_{57}ClN_2O_{11}$, 693.3724; found, 693.3758

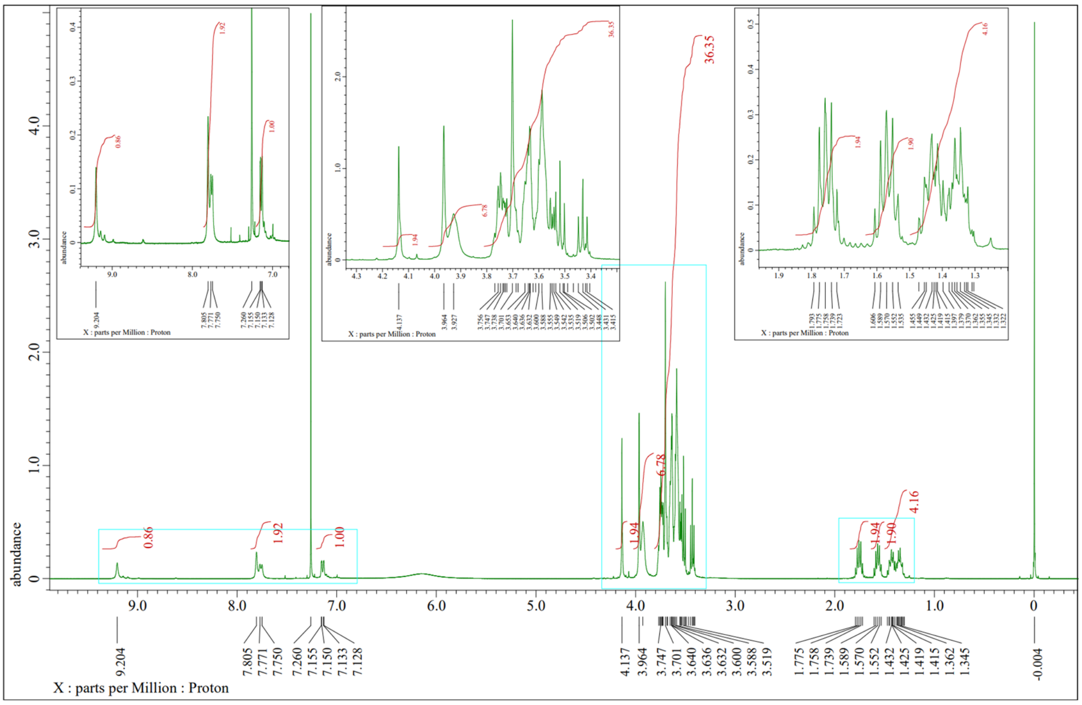

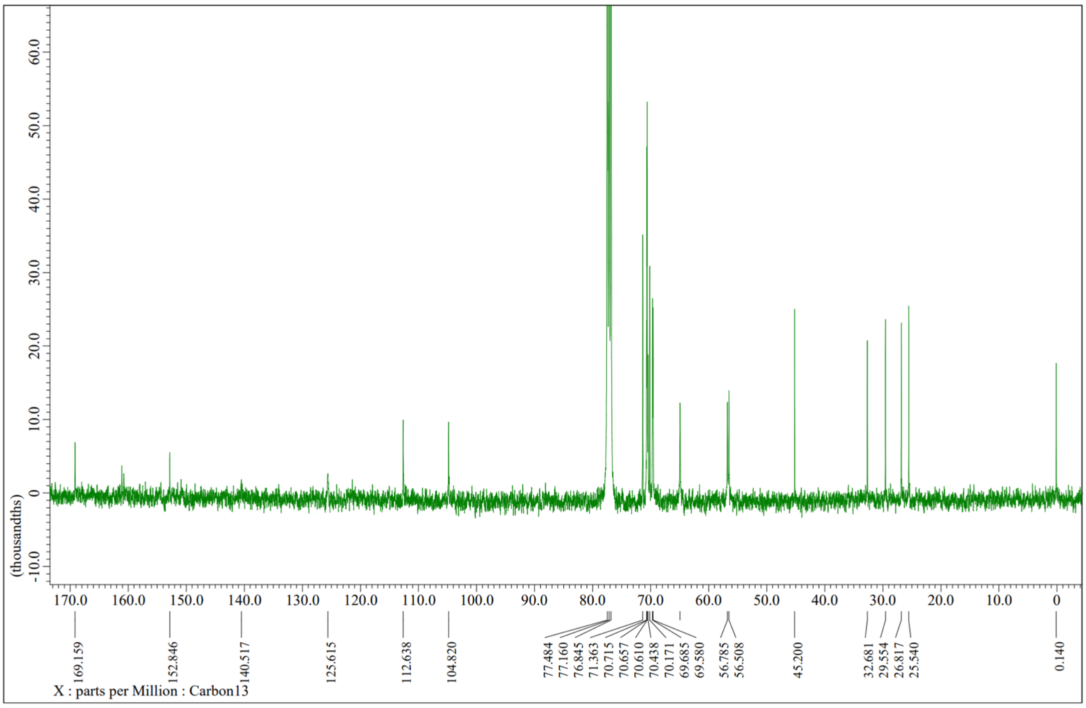

References

1 D. L. Buckley, K. Raina, N. Darricarrere, J. Hines, J. L. Gustafson, I. E. Smith, A. H. Miah, J. D. Harling and C. M. Crews, *ACS Chem. Biol.*, 2015, **10**, 1831–1837.

2 T. Schwarze, J. Riemer, H. Müller, L. John, H.-J. Holdt and P. Wessig, *Chem. – Eur. J.*, 2019, **25**, 12412–12422.

3 W. Zhu, S. Takeuchi, S. Imai, T. Terada, T. Ueda, Y. Nasu, T. Terai and R. E. Campbell, *Nat. Chem. Biol.*, 2023, **19**, 38–44.
